# Mutual Interference between Cytomegalovirus and Reconstitution of Protective Immunity after Hematopoietic Cell Transplantation

**DOI:** 10.3389/fimmu.2016.00294

**Published:** 2016-08-04

**Authors:** Matthias J. Reddehase

**Affiliations:** ^1^Research Center for Immunotherapy (FZI), Institute for Virology, University Medical Center, Johannes Gutenberg-University of Mainz, Mainz, Germany

**Keywords:** adoptive cell transfer, bone marrow stroma, CD8 T cells, Cytomegalovirus, hematopoietic cell transplantation, hemopoietins, immunotherapy, reconstitution

## Abstract

Hematopoietic cell transplantation (HCT) is a therapy option for aggressive forms of hematopoietic malignancies that are resistant to standard antitumoral therapies. Hematoablative treatment preceding HCT, however, opens a “window of opportunity” for latent Cytomegalovirus (CMV) by releasing it from immune control with the consequence of reactivation of productive viral gene expression and recurrence of infectious virus. A “window of opportunity” for the virus represents a “window of risk” for the patient. In the interim between HCT and reconstitution of antiviral immunity, primarily mediated by CD8^+^ T cells, initially low amounts of reactivated virus can expand exponentially, disseminate to essentially all organs, and cause multiple organ CMV disease, with interstitial pneumonia (CMV-IP) representing the most severe clinical manifestation. Here, I will review predictions originally made in the mouse model of experimental HCT and murine CMV infection, some of which have already paved the way to translational preclinical research and promising clinical trials of a preemptive cytoimmunotherapy of human CMV disease. Specifically, the mouse model has been pivotal in providing “proof of concept” for preventing CMV disease after HCT by adoptive transfer of preselected, virus epitope-specific effector and memory CD8^+^ T cells bridging the critical interim. However, CMV is not a “passive antigen” but is a pathogen that actively interferes with the reconstitution of protective immunity by infecting bone marrow (BM) stromal cells that otherwise form niches for hematopoiesis by providing the structural microenvironment and by producing hematopoietically active cytokines, the hemopoietins. Depending on the precise conditions of HCT, reduced homing of transplanted hematopoietic stem- and progenitor cells to infected BM stroma and impaired colony growth and lineage differentiation can lead to “graft failure.” In consequence, uncontrolled virus spread causes morbidity and mortality. In the race between viral BM pathology and reconstitution of antiviral immunity following HCT, exogenous reconstitution of virus-specific CD8^+^ T cells by adoptive cell transfer as an interventional strategy can turn the balance toward control of CMV.

## Clinical Impact of Cytomegalovirus Infection

Human Cytomegalovirus (hCMV) is the prototype member of the subfamily *Betaherpesvirinae* of the *Herpesviridae* [reviewed in Ref. (1)]. Productive primary infection of adult, immunocompetent individuals is efficiently controlled by innate and adaptive immune recognition, so that the infection usually goes unnoticed or, in the worst case, with mild and unspecific symptoms of an infectious mononucleosis-like manifestation rarely diagnosed as a manifestation of hCMV infection [reviewed in Ref. (2)]. While virus replication is terminated and viral histopathology leading to overt organ disease is prevented, replication-competent hCMV genomes persist for the lifetime of the host in cells of the myeloid hematopoietic lineage, and presumably also in endothelial cells, in a non-productive state referred to as “latency.” Presence of hCMV-specific antibodies, so-called CMV “seropositivity,” is indicative of latent hCMV infection of otherwise healthy individuals. The establishment of latency is a feature common to herpesviruses. By definition, “latency” is characterized by the absence of infectious virions, but competence to reactivate (3). As reviewed recently by Poole and Sinclair under the figurative title “Sleepless Latency of Human Cytomegalovirus” (4), latency does not imply a genome-wide transcriptional quiescence; instead, the expression of a limited set of latency-associated microRNAs, coding transcripts, and proteins manipulates host cell functions [for further reviews, see Ref. (5, 6)].

Interest in hCMV as a medically relevant human pathogen is based on severe multiple organ disease that infection can cause in the immunocompromised host, including congenital hCMV infection of the embryo/fetus, which, after the advent of vaccination against Rubella, has become the most frequent viral cause of birth defects [reviewed in Ref. (7, 8)]. Besides patients with hereditary or acquired immunodeficiencies and patients with sepsis-associated immunosuppression, patients with iatrogenic immunosuppression are a major risk group at all medical centers. This includes recipients of solid organ transplantation (SOT) and of hematopoietic cell (HC) transplantation (HCT), in which latent virus can reactivate to productive infection under the conditions of therapy-inherent immunosuppression. In SOT, ischemia/reperfusion injury and prophylaxis against graft rejection (host-versus-graft reaction) can trigger and/or facilitate virus reactivation (9, 10). In HCT, hemato-/immunoablation, prophylaxis against graft-versus-host disease (GvHD) in case of allo-HCT, and also the underlying hematopoietic malignancy itself can trigger and/or facilitate virus reactivation. Reactivation can occur within the transplant in case of a latently infected, “seropositive” donor (D^+^) or in the organs of a latently infected, “seropositive” recipient (R^+^) or in both (D^+^R^+^) [for a synopsis of clinical aspects of CMV diseases, see Ref. (2, 11, 12)].

## Why a Mouse Model? Validity of Models, Predictive Value, and Limitations of Models to Keep in Mind

Research on hCMV in human cells and tissues is limited to cell and organ culture models, observational clinical studies, biopsy and autopsy specimens, and, more recently, humanized mouse models with human tissue implants. For studying *in vivo* pathogenesis, the humanized mouse models are closest to human CMV disease and are undoubtedly instrumental [for a review, see Ref. (13)], but they also have limitations to keep in mind. As a more technical aspect, these models are demanding in terms of reaching statistical significance; yet, this can be solved by investment of resources. More critically, cytokine communication across cells from different species can be disturbed, virus spread is limited to take place between hematopoietic lineage cells and tissue implants while spread throughout the organism is interrupted, and disease cannot be studied in the context of the entirety of functional organs. This makes it impossible to study, for instance, survival benefit from antiviral therapies.

The strict host species-specificity of CMVs prevents studying hCMV in animal models (14–16). In the author’s personal view, this is not a disadvantage. CMVs have co-speciated with their respective hosts, and each CMV species has evolutionarily acquired a set of “private” genes, not shared with other CMV species, to specifically adapt to its host [reviewed in Ref. (1, 17)]. This co-speciation is associated with “biological convergence.” This means that different CMV species have evolved similar host adaptations, although arriving there by different genes and mechanisms. As a prominent example, all CMVs have evolved genes to subvert immune recognition by interfering with the cell surface presentation of antigenic peptide-loaded MHC molecules for T-cell recognition or by downregulating or expressing ligands of activatory and inhibitory natural killer (NK) cell receptors, respectively [for reviews, see Ref. (18–20)]. Thus, even if it were possible, it would make no sense to study heterologous CMV–host infections.

Most frequently used animal models are the mouse model in all its genetical variations, the guinea pig model with its advantage in addressing congenital infection [reviewed in Ref. (21)], and the rat model with its advantage in studying SOT and vascular diseases [reviewed in Ref. (22)]. Non-human primate models are considered closest to hCMV infection of humans [for a review, see Ref. (23)]. However, these models are demanding in many obvious aspects, and manipulation of host genetics as a tool of analysis is not a realistic option, unlike in the mouse model where inbred strains exist and where a host of transgenic/knock-in/knock-out strains are already available or can be made with relative ease [for a review, see Ref. (24)]. Furthermore, even non-human primate CMVs can critically differ genetically and phenotypically from hCMV (1, 23). Most importantly, one can provocatively state that “hCMV is not a model for hCMV,” as different strains of hCMV, in particular cell culture-adapted “laboratory strains” like AD169 as opposed to direct isolates from clinical samples, can differ substantially in their cell tropism and pathogenetical potential [discussed in Ref. (25)]. In fact, in terms of broad cell tropism, replicative potential, and *in vivo* pathogenicity, murine CMV (mCMV) is phenotypically closer to “clinical” isolates of hCMV than is hCMV strain AD169 on which hCMV research was focused for a long time.

Nonetheless, in the author’s view, mouse models must be carefully planned to appromixate a clinical correlate as close as feasible, and they receive their legitimation by addressing questions that cannot be addressed, or at least not easily be addressed, by clinical investigation. I leave it without referencing that many published mouse models do not comply with this aim and remain artificial. Immunotherapy of CMV disease by adoptive transfer of viral epitope-specific CD8^+^ T cells, however, is a success story in that early predictions from the mouse model [(26–28), reviewed in Ref. (29, 30)] have proven valid in clinical trials [(31–36), reviewed in Ref. (12)] and have thus survived the “test of time.” Another prediction made by the mouse model has been a preeminent antigenicity, immunogenicity, and protection-inducing capacity of immediate-early (IE) proteins, in particular of major IE (MIE) protein IE1 [(37–40), reviewed in Ref. (18)] that is expressed under the control of a strong MIE promoter–enhancer element (41). Later, the IE1 epitope of mCMV was found to be expressed during viral latency for sensing by patrolling CD8^+^ T cells [(42–44), reviewed in Ref. (45)] and was the first epitope to be identified as an inducer of “memory inflation” during viral latency [(46, 47), reviewed in Ref. (48, 49)]. Although human CD8^+^ T-cell responses against IE1 of hCMV were reported soon thereafter (50), the prediction by the mouse model was long neglected, as the human immune response to hCMV was found to be dominated instead by epitopes of the virion tegument protein pUL83/pp65 (51, 52). This difference has been used for some time to question the validity of the mouse model. We know today that clonal selection in cell culture by pUL83/pp65 abundantly present in dense body-rich virus preparations (53) used for T-cell restimulation had selected against IE-specific T cells. With the advent of direct quantitation of epitope-specific T cells using peptide libraries or MHC–peptide multimers, the MIE locus was recognized as a coding hot spot for antigenicity and immunogenicity also in hCMV infection (54–56). More recently, as a further approximation of the model to its clinical correlate, cytoimmunotherapy using human T cells, virus epitope-specific lines as well as human TCR-transduced cells, has been documented in HLA-transgenic mice infected with an antigenically “humanized” recombinant mCMV encoding an hCMV epitope. This approach allowed studying the resolution of organ infection and the demonstration of survival benefit (57).

All in all, with critical awareness of the genetic differences in both virus and host, the mouse model can make valuable predictions and define questions for an aimed subsequent evaluation in other models as well as by clinical investigation.

## Interference of CMV Infection with T-Cell Reconstitution after HCT

### Lethality of CMV Infection Post-HCT Is Determined by the Degree of Hematoablation and the Number of Transplanted HC

**Figure 1:**
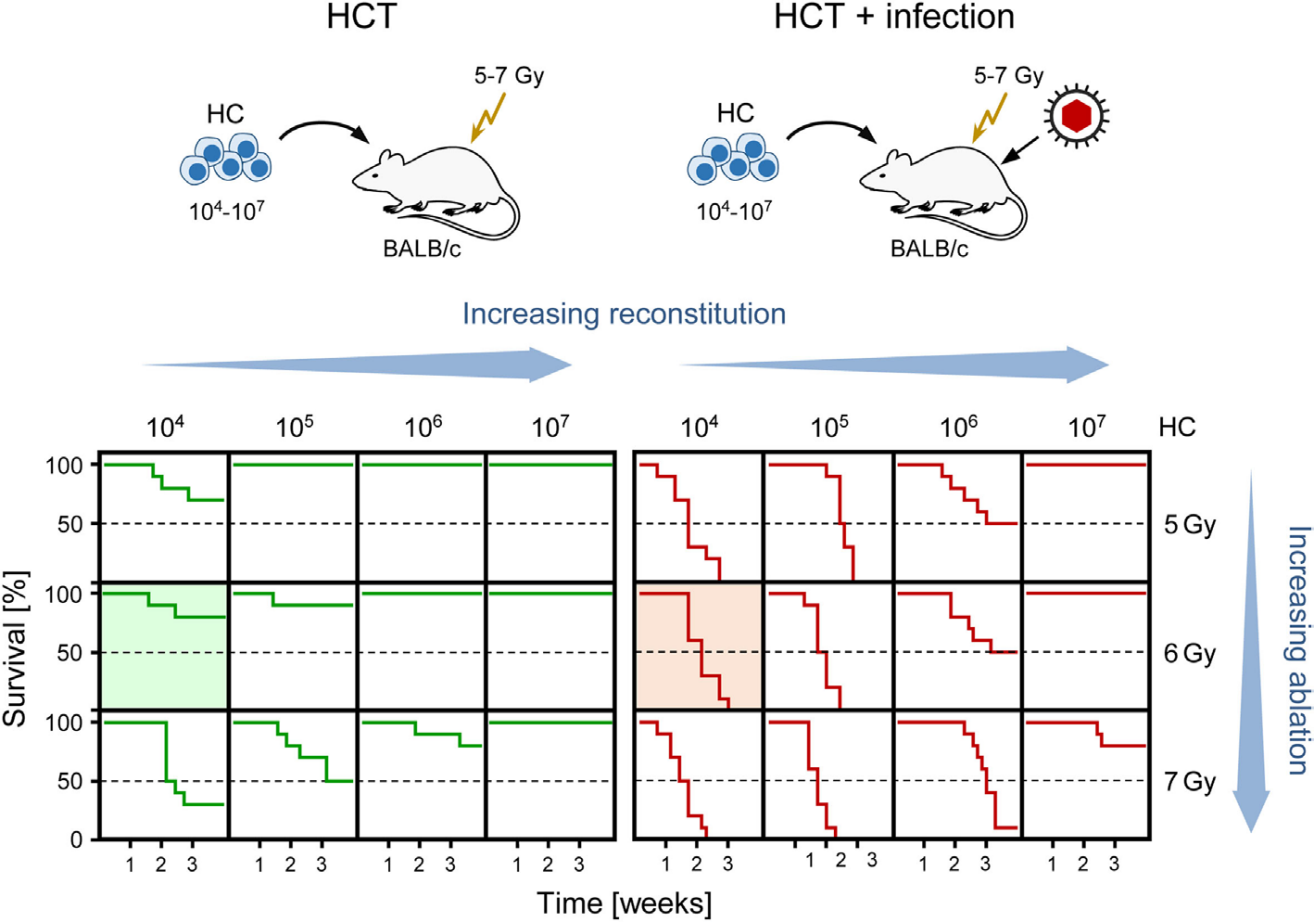
**Negative impact of CMV infection on survival rates after HCT demonstrated in the murine model**. (Top) Sketch of the experimental model, transplanting increasing numbers of hematopoietic cells (HC) to BALB/c recipients immunocompromised by hematoablative total-body γ-irradiation with single doses increasing from 5 to 7 Gy. (Bottom) Kaplan–Meier survival curves depending on the two variables HC dose (increasing reconstitution) and dose of γ-irradiation (increasing ablation). (Green lines) HCT, no infection. (Red lines), HCT and intraplantar infection with a constant dose of purified mCMV. Green- and red-shaded panels correspond to the bone marrow histology shown in Figures 3A,B, respectively. Reproduced from Ref. (59) with permission from Caister Academic Press, Norfolk, UK.

Clinical manifestations of CMV infection are difficult to predict even when infection is diagnosed by routine quantitative PCR screening. Nevertheless, virus replication indirectly defined by viral load in terms of genome copy numbers has positive predictive value for CMV disease [for a discussion of load thresholds for initiating therapy, see Ref. (58)]. As CMV disease is typically linked to an immunocompromised state, it is not far to seek that the degree of hemato-/immunoablation of the HCT recipient and the efficiency of hematopoietic reconstitution by donor HC are determinants of viral load, morbidity, and mortality. Obviously, this cannot be verified experimentally in HCT patients and would be logistically highly demanding to test in non-human primate models. We have therefore used the mouse model of HCT and infection with the Smith strain of mCMV [reviewed in Ref. (59)] to determine lethality of mCMV as a function of two variables, the dose of total-body γ-irradiation defining the degree of hemato-/immunoablation of the recipient and the number of transplanted HC defining the degree of hematopoietic reconstitution (Figure 1). The results document impressively that viral lethality can vary, depending on just these two parameters, between 0 and 100%. Comparison of the survival plots between HCT in the absence and presence of mCMV infection (Figure 1, left and right panel, respectively) documents a clear shift to overall higher mortality rates caused by the infection. These data also show that the Smith strain of mCMV is not replicatively attenuated but can cause lethal disease if host immune control fails. Previous work on the histopathology has shown that mortality from mCMV in the experimental HCT model results from direct viral cytopathogenicity, and not from immunopathogenicity, in multiple organs (60, 61).

Lethality determined by these two variables is still a reductionistic approach, as in clinical reality many more parameters contribute to determining the outcome. These include donor and host genetics, time and site of virus reactivation, virus reactivation incidence defining the effective virus dose, difference in virus strains, toxicity of a preceding cytostatic leukemia therapy, GvHD prophylaxis, and antiviral drug therapy with myelosuppressive and nephrotoxic side effects. As a consequence, the clinical outcome is “individual fate” and very difficult to predict.

### CMV Infects Bone Marrow Stroma Cells and Inhibits Myelopoiesis in Cell Culture

**Figure 2:**
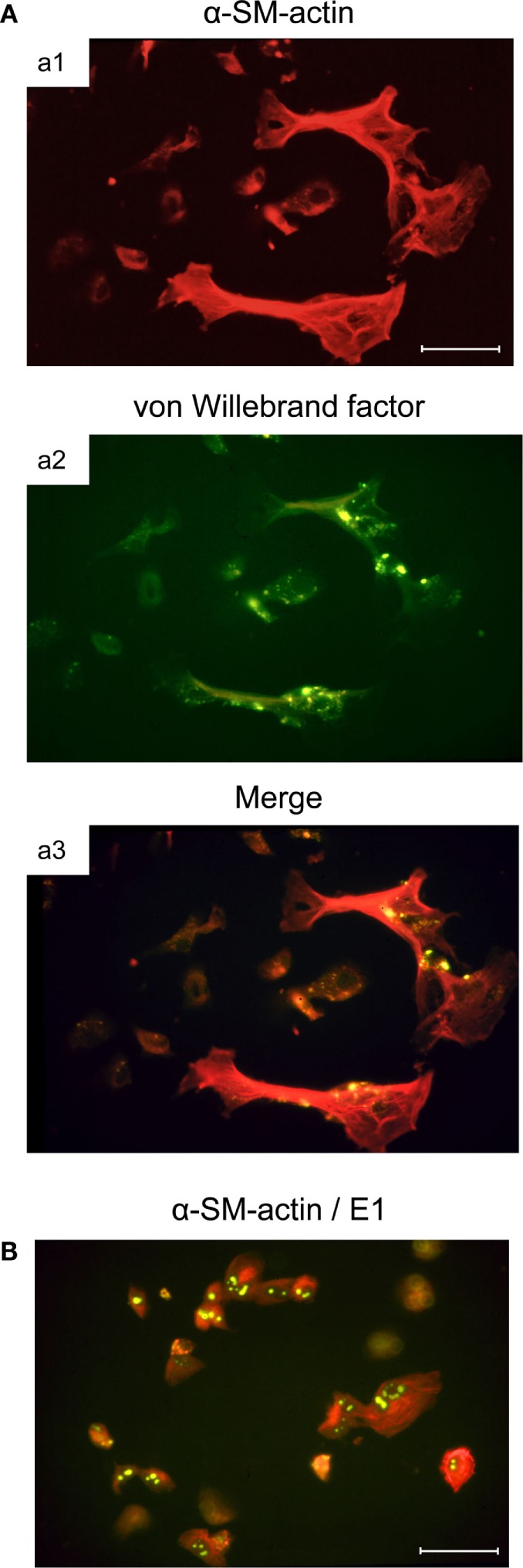
**Infection of BM stromal cells in primary cell culture**. **(A)** Images of the typically flattened, uninfected myofibroblastic BM stroma cells co-expressing smooth muscle cell marker α-SM-actin (a1, red fluorescence) and endothelial cell marker von Willebrand factor concentrated in Weibel–Palade bodies (a2, green fluorescence). (a3) merge. **(B)** Cytopathic effect of mCMV infection in the BM stroma cells. Detection of intranuclear E1 protein (spotty green fluorescence) indicates that infection has proceeded to the early (E) phase of the viral replicative cycle. Bar markers: 10 μm. Images illustrate information reported in Ref. (59).

Murine bone marrow (BM) stromal cells in primary cell culture are positive for alkaline phosphatase, while being negative for acidic phosphatase. They not only share properties with fibroblastic cells, such as the expression of fibronectin, but also express vimentin shared by mesenchymal cells, including fibroblasts, endothelial cells, and smooth muscle cells, as well as desmin, relating them to smooth muscle cells as well. Notably, they resemble myofibroblastic cells in that they express α-smooth muscle actin (α-SMA), but simultaneously also show characteristics of endothelial cells by expressing von Willebrand factor (vWF) in Weibel–Palade bodies (Figure 2A, illustrating co-expression of α-SMA and vWF in large, flattened BM-derived stromal cells). Cell surface phenotyping (not comprehensive) revealed expression of MHC-I but not MHC-II, CD4 but not CD3 or CD8, macrophage-associated molecules Mac2 and CD11b but not Mac3, and expression of CD44 and CD71 (59). This phenotype indicates a multilineage progenitor nature of the BM stromal cells [reviewed in Ref. (62)]. Importantly, these cells are susceptible to productive mCMV infection and show the cytopathic effect of detachment and rounding due to cytoskeleton disruption (Figure 2B).

Previous work has revealed that these cells support myeloid lineage hematopoiesis (generation of granulocyte–monocyte progenitors from stem cells) in long-term BM cultures (LTBMC) when uninfected. In contrast, mCMV (Smith strain) infection of LTBMC, containing an established stroma cell monolayer, leads to cessation of myelopoiesis, concominant with the peak of virus productivity and cytopathic effect in the stromal cells (63). Importantly, transfer of HC from these infected LTBMC to uninfected stroma cell monolayers rescued myelopoiesis. This finding revealed a maintained myelopoietic potential of HC recovered from infected cultures, and thus mechanistically localized the inhibition of myelopoiesis, the so-called “myelosuppression,” to infection of the stromal cells.

These *in vitro* findings for mCMV are in good accordance with an earlier study on hCMV by Apperley and colleagues (64) concluding that stromal cells, but not HC, are infected by the attenuated, high-passage fibroblast-adapted hCMV laboratory strain AD169 and, remarkably, also by four out of four tested low-passage “clinical” isolates. As this study did not involve rescue of HC from infected cultures by transfer to uninfected stroma cell monolayers, and because latent infection of HC may have remained undetected by the methods available at that time, it was open to question if virus-exposed human HC retained their full myelopoietic potential. The mouse model would predict in retrospect that this was likely the case.

The issue of the mechanism of myelosuppression by hCMV was resumed by Simmons et al. in the group of Torok-Storb by considering perturbation of stromal cell function versus direct infection of committed myeloid progenitors in human LTBMC (65). Testing laboratory strain AD169 in comparison to low-passage hCMV isolates, the authors found that 12 out of 20 isolates behaved like AD169 in that they failed to infect HC but inhibited myelopoiesis in human LTBMC associated with infection of stromal cells, whereas the remaining 8 isolates showed tropism for hematopoietic progenitors. Notably, four out of these eight isolates showed little tendency to infect the stromal cells, indicating loss of stromal cell tropism and acquisition or maintenance of HC tropism.

In the light of abundant evidence for latent infection of hematopoietic stem and/or progenitor cells by hCMV (66–70), it would be of interest if this really applies to all isolates of hCMV or if it might possibly correlate with the tropism differences reported for the mechanisms of myelosuppression. To our knowledge, virus reactivated *in vitro* from highly purified, contaminant-free, latently infected HC of otherwise healthy volunteers has not been typed and tested for its cell tropism. For reactivation *in vivo*, that is in patients in whom hCMV has reactivated, it is difficult, if not impossible, to unequivocally identify the cellular site of the reactivation event. As low-passage isolates from patients with CMV disease usually result from recent reactivation events, and as most of those do not show HC tropism (see above), it is likely that they were derived from non-HC cell types. This would also explain why the highest risk of reactivation in HCT recipients is associated with latent hCMV carriage by the recipient (R^+^), whereas it is associated with latent hCMV carriage by the donor (D^+^) in SOT (71). Of interest in this context is the finding that in a human–sheep xenograft model human hematopoietic stem cells (HSCs) that mediate long-term *in vivo* engraftment were not susceptible to hCMV infection (72). Likewise, in the mouse model, HCT performed with BM cells from latently infected donors failed to transmit latent mCMV to recipients. This finding indicated that hematopoietic stem and/or progenitor cells that repopulate the BM did not carry and multiply latent viral genome upon their proliferation and differentiation, whereas donor organs (lungs, liver, and spleen) at the same time harbored reactivation-competent latent virus at a high load (73).

The difference between mCMV and hCMV regarding latency in HC is currently interpreted as a fundamental pathobiological difference between the two viruses and is taken as an argument against the mouse model. One must consider the possibility, however, that hCMV variants with stroma cell tropism will not be detected in HC and therefore remained unreported in the past. Such variants are functionally more analogous to mCMV than are the variants with tropism for HC.

With today’s advanced knowledge of molecular and phenotypic hCMV strain differences, in particular in the expression of the virion envelope glycoprotein complexes involved in cell entry and thus in cell tropism, namely gH/gL/gO and gH/gL/UL128–131A, the latter of which is lost upon high passaging [reviewed in Ref. (25, 74)], it would be of interest in retrospect to relate expression of these complexes to tropism for HC and stromal cells. To our knowledge, this has not been tried yet. We consider it unlikely, however, that two-thirds of the low-passage isolates studied by the group of Torok-Storb were negative for UL128–131A *ab initio* or had lost these genes upon short-term propagation in cell culture. Importantly, whereas the designation “low passage” does not exclude mutations compared to the virus present in the original clinical sample, as it was critically discussed recently by Wilkinson and colleagues (25), Simmons et al. reported to have performed their experiments with isolates derived from clinical samples after just two or three passages in human foreskin fibroblasts (65). Although even such a short-term propagation, which is technically unavoidable for doing experiments, does not formally exclude adaptation by mutations *in vitro*, it appears unlikely that 12 out of 20 independent isolates have lost HC tropism so rapidly in cell culture. One must consider, however, that the selection of mutations, as they occur in cell culture, may likewise occur *in vivo* during spread and high replication of reactivated virus in non-hematopoietic host tissue cells (fibrocytes, endothelial cells, and diverse epithelial cell types) following hematoablative treatment of an HCT recipient. So, virus in “fresh” clinical samples likely has already undergone many rounds of replication in the patient during which adaptation to non-HCs in host tissues may have occurred unnoticed.

Like hCMV, mCMV expresses alternative gH/gL envelope complexes, namely gH/gL/gO and gH/gL/MCK-2 (75). As we have shown recently, gH/gL/gO is essential for initiating infection of liver macrophages, liver sinusoidal endothelial cells (LSEC), and hepatocytes, whereas gH/gL/MCK-2 is non-essential in the presence of gH/gL/gO but can substitute for missing gH/gL/gO in intra-tissue cell-to-cell spread as well as in the infection of salivary gland tissue (76).

It was recently recognized that the prototypic bacterial artificial chromosome (BAC)-cloned virus MW97.01 and viruses with mutations introduced into the parental BAC plasmid pSM3fr by site-directed mutagenesis (77–79) express a truncated variant of MCK-2 due to a single nucleotide deletion polymorphism in the coding sequence (80). As the ATCC-distributed Smith strain VR194, with which the murine LTBMC infections were performed, represents a mixture of virions with full-length and short MCK-2, tropism for stromal cells and missing tropism for HC is apparently not related to gH/gL/MCK-2 requirement for cell entry.

In conclusion, with regard to the mechanism of myelosuppression in HC culture, the mCMV model is in line with most, though not all, low-passage isolates of hCMV. It is therefore an appropriate model for hCMV strains/isolates with stromal cell tropism that represent the majority of isolates analyzed under this aspect so far (65). This must be kept in mind as a limitation of the current mouse models, but on the other hand, one must also more appreciate differences between hCMV strains/isolates that in some aspects exceed the differences between CMVs of different species.

### Infected BM Stroma *In Situ* Fails to Support Repopulation of BM by Low-Dose HCT due to Stromal Hemopoietin Deficiency

As discussed above, “cell culture only” experiments should preferably be performed with the human pathogen, except to show equivalence of results between human pathogen and model pathogen, hCMV and mCMV in the here discussed case, for subsequent experimental *in vivo* studies in the animal model. Any animal “model” for human disease should be approximated to the clinical correlate and get its legitimation by experimental *in vivo* approaches to clinical questions that cannot be answered by observational clinical studies. The mouse model of experimental infection of immunocompromised HCT recipients provided “proof of principle” for HC graft failure due to a functional deficiency of infected BM stroma in providing the essential microenviromental support for HC homing, HSC self-renewal, and hematopoietic lineage differentiation (81–86). Under conditions of a sublethal degree of hematoablative treatment and low-dose HCT, most mice survive in the absence of infection but die if infected (Figure 1, shaded survival plots), which clearly shows the viral etiology of mortality in such a setting.

**Figure 3:**
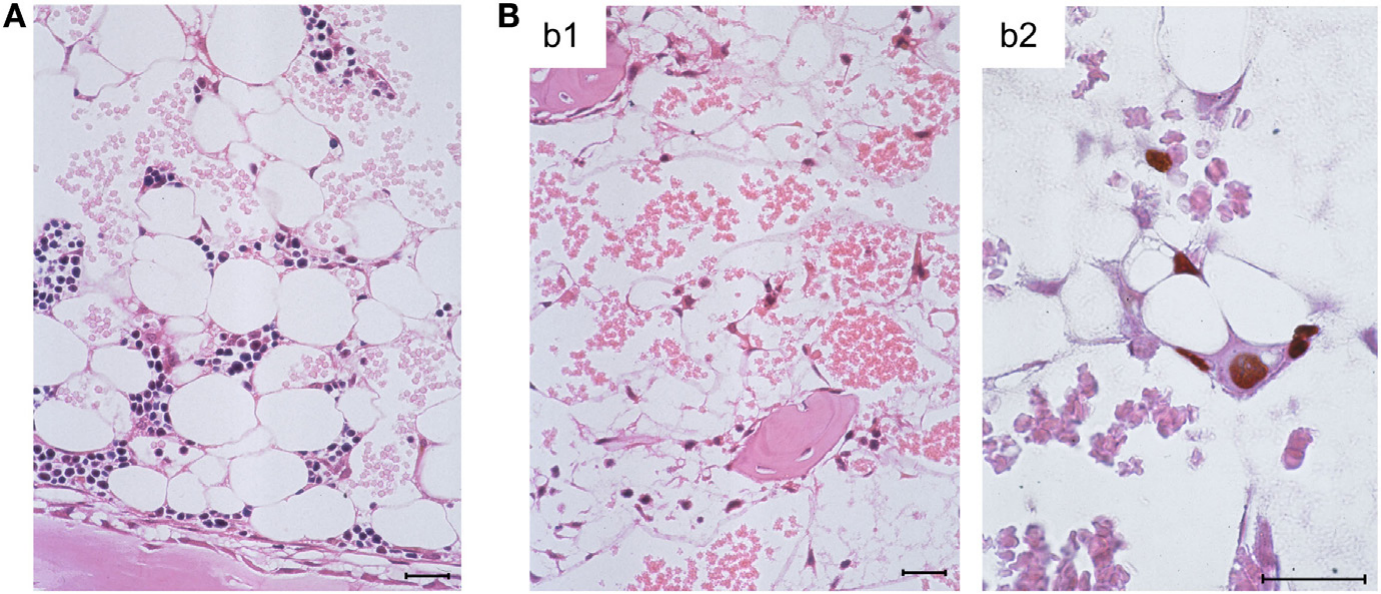
**Bone marrow aplasia caused by infection**. **(A)** Overview image of BM histology performed on day 14 after HCT, revealing a beginning repopulation of the BM of the HCT recipients with myeloid lineage colonies homing to the BM stroma in a femoral diaphysis in the absence of infection. The image corresponds to survival shown in Figure 1, green-shaded panel. **(B)** Images document absence of hematopoiesis in infected HCT recipients under conditions otherwise identical to those in **(A)**, corresponding to high mortality shown in Figure 1, red-shaded panel. (b1) Overview, showing empty stromal network in a femoral epiphysis. (b2) Detail, with immunohistological staining of viral intranuclear protein IE1 (brown staining), detecting *in situ* infected, network-forming stromal cells. Bar markers: 25 μm, throughout. Note that the size and shape of the stromal cells *in situ* matches their size and shape in cell culture (compare Figure 3B, b2 with Figure 2A). Images reproduced in rearranged form from Ref. (59, 84, 86) with permissions from Caister Academic Press, Norfolk, UK and from the *Journal of Virology*, American Society for Microbiology.

Histological images in these groups revealed BM-repopulating hematopoietic colonies, including myelomonocytic and erythroid sublineage colonies of the myeloid lineage (86), in the absence of infection compared to complete BM aplasia in the presence of infection (Figures 3A,B, b1, respectively). It should be noted that BM aplasia is also reflected by a pancytopenia, including thrombocytopenia, in the blood, and by petechial bleedings. This situation is reminiscent of thrombocytopenic purpura in newborns with congenital CMV disease (8, 18). Network-forming stromal cells are infected in the BM, as shown by immunohistochemical (IHC) staining for intranuclear IE1 protein, though the stromal network does not seem to be texturally disrupted (Figure 3B, b2). In accordance with these findings, stromal gene expression included transcripts for viral regulatory protein m123/IE1 and the late envelope glycoprotein M55/gB, thus confirming stromal infection (84). Very sensitive detection of infectivity in infected BM by a cell culture assay for IE1 gene expression (RT-PCR) transferable to permissive mouse embryo fibroblasts as indicator cells revealed a low-level productive infection of BM stroma (86). In both parameters, IE1 transcripts and infectivity, stromal infection was not notably reduced by HCTs performed with increasing numbers of transplanted HC (86), which indicated that transplanted HC do not exert a stroma-protective, innate antiviral function. Unaltered levels of transcripts from cellular housekeeping genes *pthrp* and β*-actin* reflected comparable numbers of stromal cells in both groups in accordance with the virtually intact stromal network (84). Notably, however, transcripts for the hemopoietins stem cell factor (SCF, also known as *Kit*-ligand or *Steel* factor), granulocyte colony-stimulating factor (G-CSF), and interleukin-6 (IL-6) were significantly reduced upon infection of BM stroma (84–86).

As stromal cytokines operate as growth and differentiation factors for HSCs and lineage-committed progenitor cells [for a review, see Ref. (87)], this functional deficiency of the stroma can explain the reduced hematopoiesis. It should be noted that these early studies on hemopoietin gene expression were not comprehensive, and revisiting this issue with differential high-density microarray analyses likely will reveal more alterations in stromal gene expression relevant to the hematopoiesis-supporting function of BM stroma, possibly also beyond known hemopoietins.

### High-Dose HCT Enables Partial BM Repopulation Sufficient for Surviving Infection

**Figure 4:**
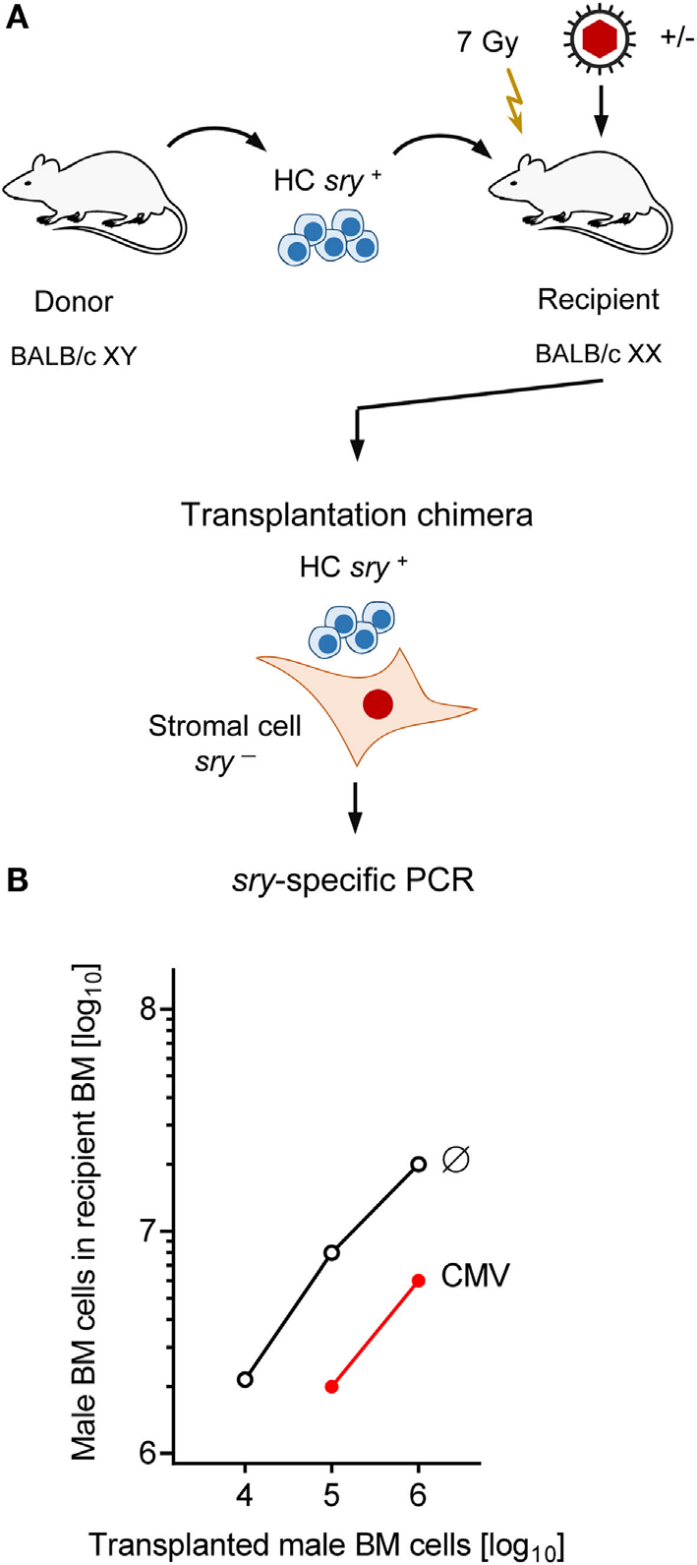
**Infection inhibits HC engraftment**. **(A)** Sketch of the experimental design of transplanting male (XY, gene *sry*^+^) HC to female (XX, gene *sry*^−^) BALB/c recipients, resulting in transplantation chimeras with *sry*^+^ HC and *sry*^−^ stromal cells. Flash symbol: total-body γ-irradiation **(B)** PCR quantitation of *sry*^+^ HC engraftment on day 14 after HCT without infection (Ø) or with infection (CMV). Modified from Ref. (86) with permission from the *Journal of Virology*, American Society for Microbiology.

The survival plots (Figure 1) made evident that mCMV infection in experimental HCT settings is not inevitably lethal but that mortality can be prevented by high-dose HCT performed with a sufficiently high number of transplanted HC, and thus of HSC. Long-term survival implied a successful repopulation of BM and control of the infection. It remained open to question, however, if BM stroma pathogenesis of mCMV is prevented by high-dose HCT or is still operative, though with an incomplete inhibition of hematopoiesis. The latter possibility proved to be correct in an approach of transplanting graded numbers of male (XY) genotype *sry*^+^ donor HC into female (XX) genotype *sry*^−^ recipients, thus generating chimeras with *sry*^+^ HC and *sry*^−^ stromal cells (Figure 4A) (86).

For a better understanding of this approach, it is important to recall that BM stromal cells present in the donor BM cell population are not transplantable under conditions of HCT and thus do not home to the recipient’s BM compartment [reviewed in Ref. (62)]. We have experimentally reconfirmed this previously by a serial transplantation of female donor BM cells into male recipients, leading to chimeras with *sry*^−^ HC and *sry*^+^ stromal cells, followed by transplantation of the chimera-derived BM cell population into female *sry*^−^ secondary recipients. The *sry* gene of the chimeras’ stromal cells was not detectable by sensitive PCR in the BM of the secondary recipients (84).

Quantitation of BM-repopulating *sry*^+^ HC in the XY–XX chimeras by *sry* gene-specific PCR revealed a low level of BM repopulation by HCT performed with 10,000 HC, and its mortality-associated extinction by mCMV infection (Figure 4B). Importantly, although increasing doses of transplanted HC led to detectable progeny repopulating the BM to an increasing degree even after mCMV infection, an inhibition became apparent for all tested doses, as indicated by parallel log–log linear input-versus-output graphs (86). Note that repopulation based on 10^5^ donor HC was not sufficient for survival in the presence of infection, whereas repopulation based on 10^6^ or more donor HC led to increasing survival rates (compare repopulation data in Figure 4B with survival rates in Figure 1).

**Figure 5:**
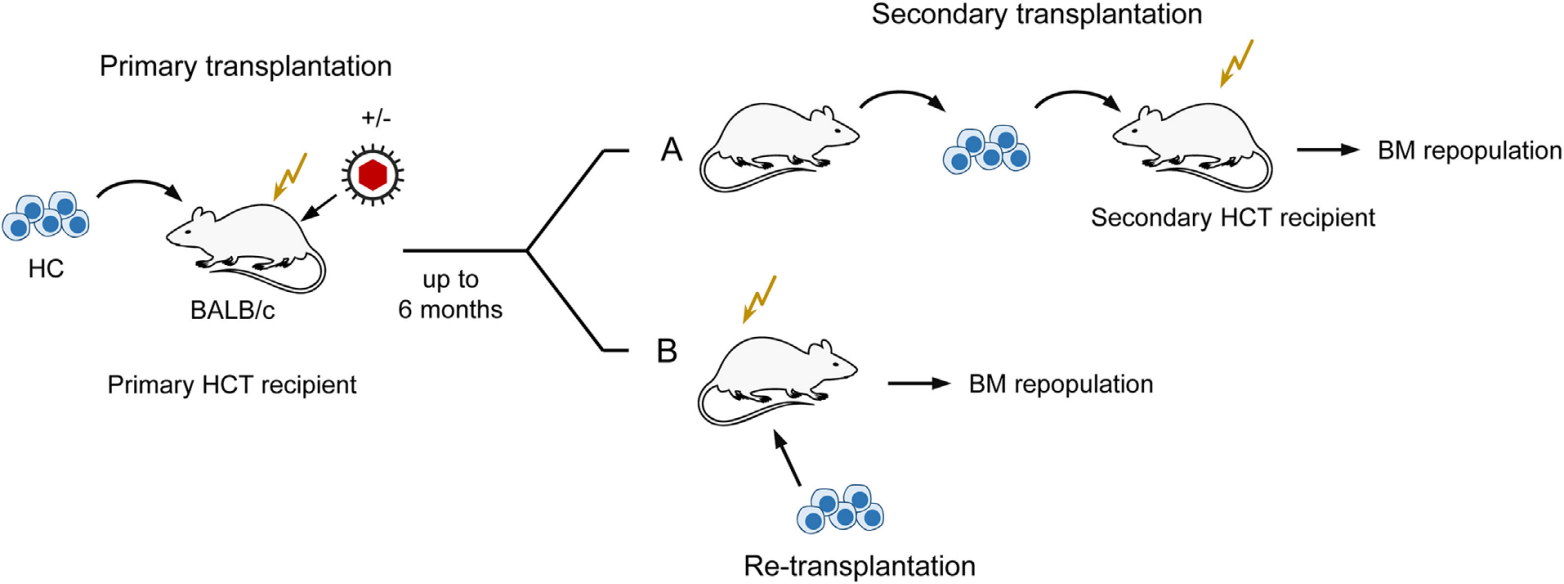
**Sketch of serial transplantation and re-transplantation protocols**. **(A)** Serial transplantation of HC for quantitating BM-repopulating hematopoietic stem cells (HSC) depending on a past productive infection of primary HCT recipients. Infected primary HCT recipients become lastingly deficient in hematopoiesis reflected by reduced numbers of HSC. **(B)** Localization of the cause of enduring hematopoietic deficiency in primary HCT recipients with a past productive infection. Failure to cure by re-transplantation with normal HC localizes the hematopoietic deficiency to the BM stroma. This Figure illustrates the experimental regimens used to demonstrate enduring bone marrow stroma deficiency reported in Ref. (82, 83).

Notably, the initial stromal damage set by the acute infection of HCT recipients was not repaired after clearance of productive infection and instead led to a lasting reduction (observation periods of 1, 3, and 6 months) in the number of HSC capable of self-renewal and long-term BM repopulation (82, 83). This was shown by a limiting dilution quantitation of HSC present in the BM of uninfected compared to infected primary HCT recipients by serial transfer of their HC in graded numbers into uninfected secondary HCT recipients, followed 6 weeks later by monitoring of BM repopulation by donor HC (for a sketch of the experimental regimen, see Figure 5A). Specifically, when HC were derived from latently infected compared to uninfected primary HCT recipients as donors for serial transfer, five-times more HC, and thus HSC, were needed to long-term repopulate the BM of uninfected secondary HCT recipients. Notably, this lastingly reduced HSC frequency in the BM of latently infected primary HCT recipients was not caused by a latent infection of HC/HSC, as one might have surmised, but could be attributed to an irreversible deficiency in stromal function, as it was not cured by re-transplantation with fully functional HC from untreated, normal donors (83) (Figure 5B). Altogether, although high-dose HCT allows BM repopulation sufficient for a level of hematopoietic reconstitution that prevents lethal CMV disease, latently infected HCT recipients remain lastingly deficient in their stromal function and in their transplantable hematopoietic potential.

**Figure 6:**
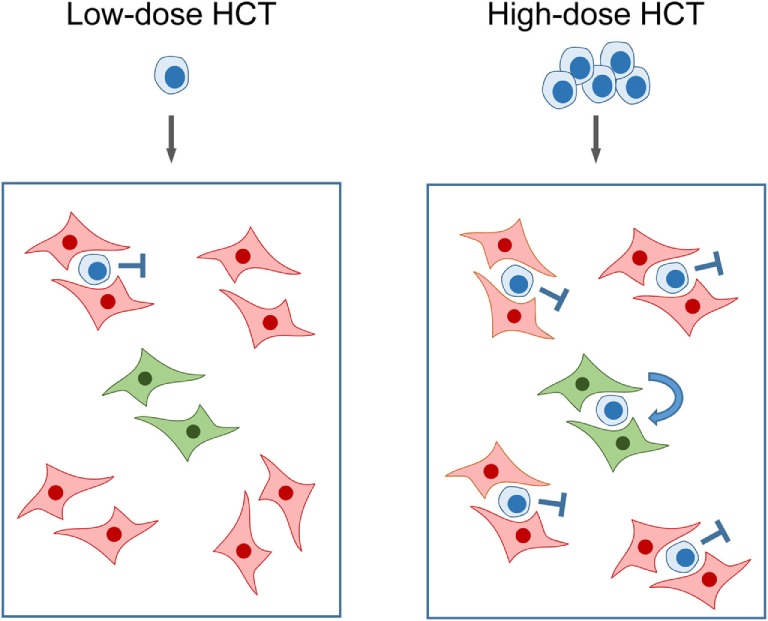
**Model of “closed” and “open” hematopoietic niches**. (Left) Low-dose HCT: in the model example, a single transplanted HSC has a 20% chance to occupy a single “open niche” (green) out of a total of five niches of which four are closed (red) due to CMV infection of the BM stromal cells that form the niche. (Right) High-dose HCT: high probability of occupying the single “open niche” out of five niches when five HSC are transferred. Note that the probability for a successful lodging event is <100% since more than one HSC may try to lodge to a “closed niche,” but the overall probability for the event that at least one out of five transplanted HSC lodges to the single “open niche” is increased. Importantly, the rate of stroma cell infection is not notably influenced by the dose of transplanted HC over the range tested. Stop symbol: hematopoiesis is not supported. Bent arrow: successful HSC lodging and self-renewal. This Figure provides a graphical new explanation for data published in Ref. (86), corresponding to Figure 4B.

Bone marrow repopulation after high-dose HCT in the absence of stroma repair clamors for an explanation: if hemopoietin expression is so in deficit that it fails to support few HSC transferred by low-dose HCT, how can it then support a higher number of HSC transferred by high-dose HCT? An explanation is provided by the model of “hematopoietic niches” (Figure 6). The existence of a BM niche, the location in BM stroma in which an HSC resides, was proposed in the end 1970s (88) and is still highly topical as indicated by several very recent review articles (89–92). In essence, a niche is defined as a microanatomical site, formed by BM stroma, which provides the physical environment and locally delivered hemopoietins for HSC lodging, self-renewal, and lineage differentiation. Important in view of reduced SCF gene expression upon stromal infection (see above) is the finding that the transmembrane-bound form of SCF, the tmSCF, is a critical hemopoietin in HSC lodgment (93). Accordingly, what counts is the local stromal support of HSC in the niche, rather than systemic hemopoietin levels. To explain our findings, we can therefore propose that partial infection of BM stroma “closes” a proportion of the niches, while other niches remain “open.” Upon low-dose HCT, the few HSC mostly meet “closed niches” with a low chance to lodge to an “open niche,” whereas the likelihood for occupying “open niches” increases with the number of transplanted HSC despite an unaltered rate of stroma cell infection (Figure 6).

### CMV-Mediated Hematopoietic Deficiency Leads to Reduced Reconstitution of CD8^+^ T Cells

**Figure 7:**
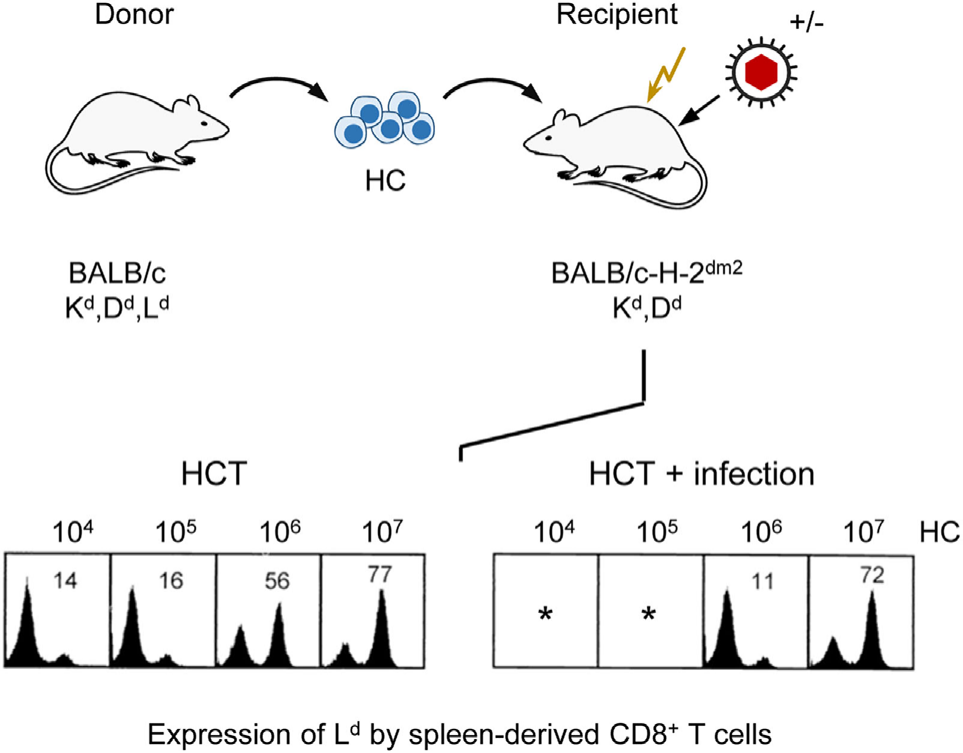
**Infection of HCT recipients impedes the reconstitution of CD8^+^ T cells**. (Top) Sketch of the experimental protocol, transplanting HC from BALB/c mice to immunocompromised (flash symbol: sublethal γ-irradiation with a dose of 6 Gy) mutant mice not expressing the MHC-I molecule L^d^. (Bottom) Cytofluorometric analysis of L^d^ expression in the recipients revealed chimerism within the spleen-derived CD8^+^ T-cell population that shifted toward donor-type reconstitution with increasing doses of transplanted HC (left panel, HCT). This shift is impeded by infection (right panel, HCT + infection). *100% mortality. Percentages of donor-derived CD8^+^ T cells are indicated. Modified from Ref. (96) with permission from the *Journal of Virology*, American Society for Microbiology.

As T lymphopoiesis is based on progenitors in the BM, hematopoiesis reduced by stromal deficiency should also impact on downstream T-cell reconstitution. As clinical data have correlated control of infection with the reconstitution of CD8^+^ T cells (94, 95), we monitored reconstitution of donor-type CD8^+^ T cells in uninfected compared to infected, sublethally irradiated, HCT recipients depending on the number of transplanted donor HC in an MHC-I disparate HCT model (Figure 7). In this model, the MHC class-I (MHC-I) molecule H-2L^d^ expressed on donor (BALB/c: K^d^D^d^L^d^) HC serves as a cytofluorometric marker for donor-type reconstitution of genetically L^d^-negative recipients (BALB/c-H-2^dm2^: K^d^D^d^) in which a graft-versus-host reaction to MHC-I is excluded by antigenic match and a host-versus-graft reaction to L^d^ expressed by the donor cells is avoided by transplantation tolerance. T cells reappear in the spleen in the third week after HCT. Based on a component contributed by radiation-resistant thymic T-cell precursors, a chimerism with donor-type (L^d^-positive) and recipient-type (L^d^-negative) CD8^+^ T cells is established in the splenic T-cell population. As expected, increasing doses of transplanted donor HC shift the chimeric balance to the donor side in uninfected HCT recipients. Notably, this “donor shift” required more donor HC when recipients were infected (96).

In conclusion, by infecting BM stroma, CMV infection interferes with the reconstitution of donor-derived CD8^+^ T cells. In can be proposed that by inhibition of the reconstitution of virus-specific CD8^+^ T cells, CMV enhances BM pathogenesis in a feed-back loop, resulting in a less efficient antiviral control and thus a prolonged virus replication, bearing a risk of CMV organ disease.

## Interference of T-Cell Reconstitution with CMV Infection after HCT

### Reconstitution of Antiviral CD8^+^ T Cells Is Essential for Controlling CMV Infection after HCT

Early immunomonitoring studies in HCT patients indicated that control of reactivated hCMV infection correlates with the reconstitution of antiviral CD8^+^ T cells (94, 95), and subsequent adoptive cell transfer studies in the mouse model (see above) as well as in clinical trials (see above) confirmed a protective antiviral function of primed CD8^+^ effector and/or memory T cells. It was therefore reasonable to propose that reconstitution of CD8^+^ T cells after high-dose HCT is the critical parameter for the control of CMV infection and survival.

On the other hand, mCMV infection models for experimental conditions other than HCT have shown that CD8^+^ T cells are not indispensable for controlling the infection. Specifically, long-term CD8^+^ (CD8^+^ T cells and CD8^+^DC)-depleted but otherwise immunocompetent mice (97) as well as β2m knock-out mice deficient in mature cell surface MHC-I and thus also deficient in CD8^+^ T cells (98) control virus replication in all organs, including salivary glands, by alternative antiviral effector mechanisms involving CD4^+^ T cells and innate immune functions. In a more recent study comparing mCMV infection control in TCR α/β and/or γ/δ deficient mice, it was concluded that γ/δ T cells are as competent as α/β T cells in protecting mice from CMV-induced death (99). It is important to recall that alternative mechanisms did not develop after short-term CD8 depletion, suggesting that a remodeling of immune homeostasis to functionally substitute for CD8^+^ T cells needs time. Dispensability of CD8^+^ T cells was also suggested by cell transfer models showing that NK cells (100), memory B cells (101), effector CD4^+^ T cells (102) as well as γ/δ T cells (99, 103) can, in principle, confer protection against mCMV infection.

However, the HCT model gave us a lesson on how careful one must be with extrapolating mechanisms of antiviral control from one experimental setting to another. Unlike the situation in long-term CD8^+^ T cell-depleted or genetically CD8^+^ T cell-deficient mice, in which an altered immune homeostasis, not involving CD8^+^ T cells, has time to develop, reconstitution in HCT patients must rapidly provide antiviral effector cells to come in time for preventing viral spread that otherwise would lead to lethal viral pathogenesis.

For studying a scenario designed as a model that more closely approximates a clinical correlate, we focused our investigation in the mouse model on the infection of the lungs after HCT, as interstitial pneumonia (CMV-IP) is the most critical organ manifestation of CMV disease in HCT patients [reviewed in Ref. (104, 105)]. As revealed by the mouse model, lungs are not only a major target organ of viral pathogenesis (26, 61, 106–109) but also a site of virus latency and reactivation (110) as well as of “memory inflation” during latency [reviewed in Ref. (48, 49)].

**Figure 8:**
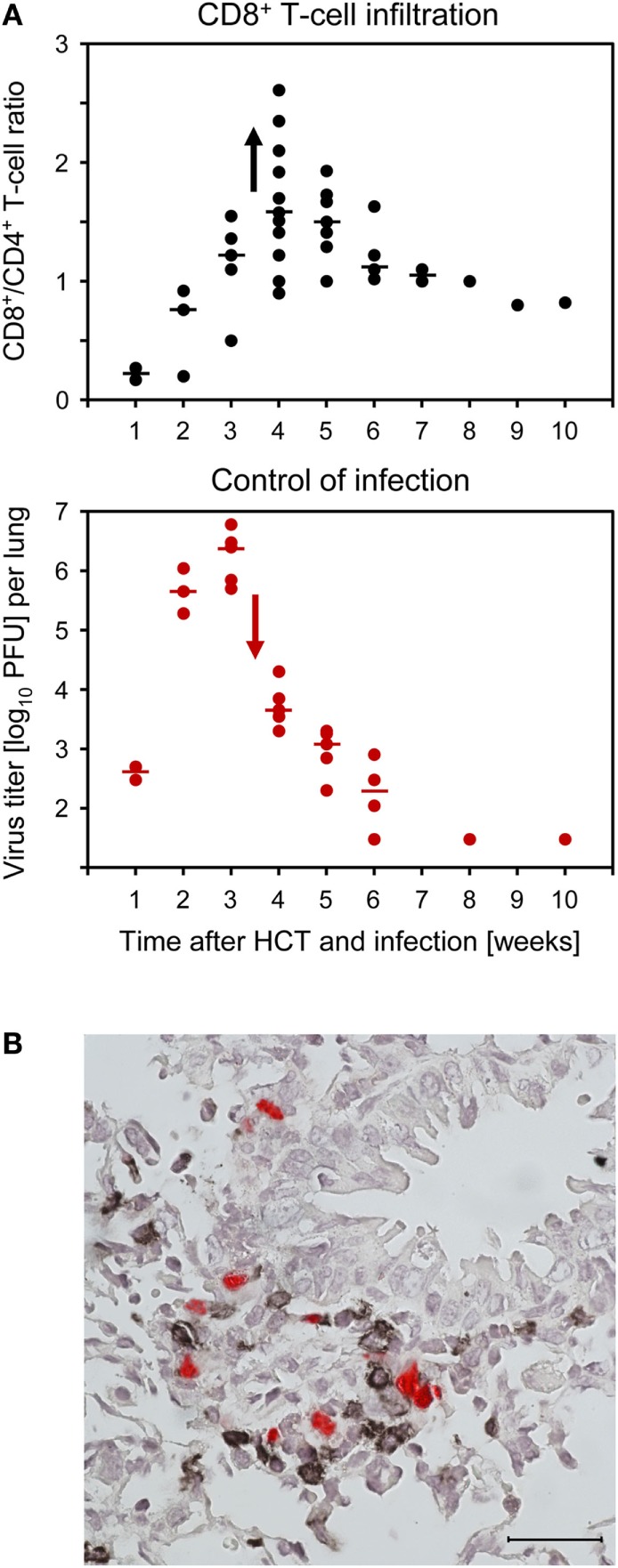
**CD8^+^ T-cell infiltration of infected lungs after HCT coincides with control of the infection in nodular inflammatory foci (NIF)**. HCT was performed with the high dose of 10^7^ HC, a condition under which infected recipients survive (recall Figure 1). **(A)** Coincidence of the peak of CD8^+^ T-cell infiltration (top panel) and the onset of decline in titers of infectious virus (bottom panel). Symbols represent data from individual mice with the median values marked. Reproduced from Ref. (59, 109) with permissions from Caister Academic Press, Norfolk, UK and from the *Journal of Virology*, American Society for Microbiology. **(B)** Confinement of pulmonary infection in NIF; shown here is a NIF with peribronchiolar localization. Two-color IHC with red-staining of IE1 protein in infected lung cells and black staining of T cells. The bar marker represents 25 μm. This image has been the cover photograph of *Journal of Virology*, volume 74, issue no. 16 (August 2000), accompanying the publication cited here as Ref. (61). Reproduced with permission from the American Society for Microbiology.

Compared to uninfected HCT recipients, lungs of infected HCT recipients showed massive leukocyte infiltration, notably including granulocytes, and widened alveolar septae (109). Infected cells in the lungs include endothelial cells of pulmonary capillaries, interstitial fibrocytes, pneumocytes of the alveolar epithelium, and alveolar macrophages. CD8^+^ T cells dominated the T-cell infiltrates in infected HCT recipients, and their peak of infiltration at 4 weeks after HCT coincided with the beginning decline in virus titers (Figure 8A). Instant *ex vivo* cytolytic activity of pulmonary infiltrate cells, not requiring *in vitro* restimulation, was mediated by TCRα/β^+^CD3^+^CD8^+^ T cells, but not by TCRα/β^+^CD3^+^CD4^+^ T cells or TCRγ/δ^+^CD3^+^ T cells (109), and cytolytically active TCRα/β^+^CD3^+^CD8^+^ T cells recovered from pulmonary infiltrates protected against CMV pathogenesis upon adoptive cell transfer (61, 96). The *ex vivo* cytolytic activity was completely inhibited by CMA (folimycin, concanamycin A) (109), known to selectively block the perforin-based pathway of cytolysis (111). More recent work has determined the predominant phenotype of the pulmonary infiltrate CD8^+^ T cells as CD62L^low^KLRG1^high^ (112), a phenotype attributed to short-lived effector cells, SLECs (113).

Typically, tissue-infiltrating CD8^+^ CTL are attracted toward infected tissue cells to form nodular inflammatory foci (NIF) (Figure 8B) (57, 61, 96, 114–116). NIF are microanatomical structures in which the infection is confined and eventually resolved; thus, NIF formation is indicative of antiviral control and protection. Importantly, NIF formation by tissue-infiltrating CTL requires the presentation of cognate epitopes on the infected tissue cells. This has been shown by transfer of cells from epitope-specific CTL lines (CTLL) and infection with viruses in which the C-terminal amino acid residue of the respective antigenic peptide (mostly a nonapeptide) is mutated to Ala, mutations X9Ala, a strategy known to largely reduce peptide processing at the steps of proteasomal cleavage, precursor peptide transport into the ER, and MHC-I binding (117). While the transferred CTLL formed NIF and protected against wild-type virus infection, the same cells did not arrange into NIF and failed to protect against X9Ala mutant viruses (57, 114).

**Figure 9:**
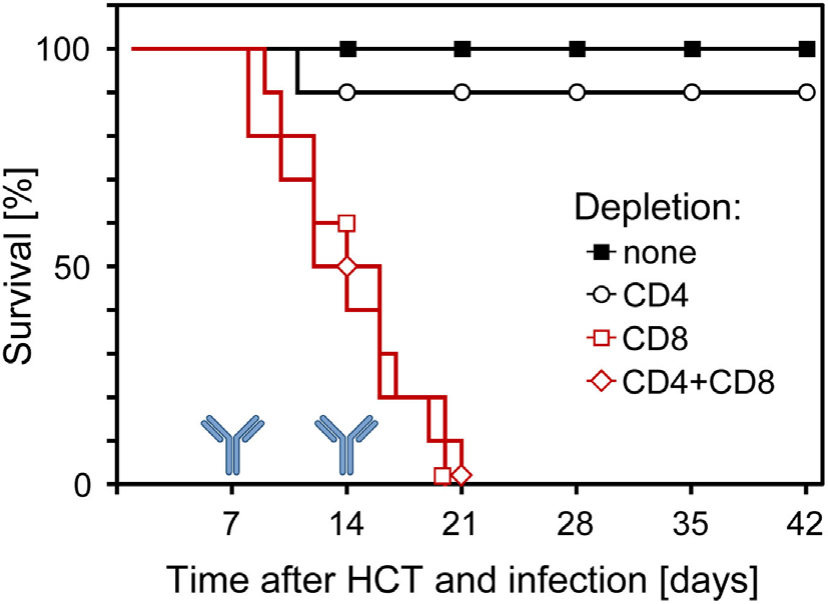
**Survival of infected HCT recipients depends on the reconstitution of CD8^+^ T cells**. In the process of an ongoing reconstitution after HCT, T-cell subsets were depleted on days 7 and 14. Shown are Kaplan–Meier survival curves that reveal 100% mortality only when CD8^+^ T cells were depleted. Reproduced from Ref. (59, 61) with permissions from Caister Academic Press, Norfolk, UK and from the *Journal of Virology*, American Society for Microbiology.

Although all this information strongly indicated a dominant role for CD8^+^ T cells in controlling CMV infection in the phase of reconstitution after HCT, it remained open to question if they are essential or if antiviral control is secured by redundance. Thus, analogous to what was found in the other models discussed above, alternative effector mechanisms might take over in case absence of CD8^+^ T cells leads to an altered immunoregulation and homeostasis. This possibility was tested by depleting T-cell subsets in infected HCT recipients on days 7 and 14 of an ongoing reconstitution. The result was unequivocal in that mortality invariably was 100% when HCT recipients were depleted of CD8^+^ T cells in the course of reconstitution, whereas almost all recipients survived infection after depletion of CD4^+^ T cells (Figure 9) (60, 61). Thus, in the specific case of an ongoing reconstitution after HCT, CD8^+^ T cells are not replaceable in their function with any other adaptive or innate immune cell type. This finding is of medical relevance, as it gives a warning against treating GvHD after allogeneic (minor histocompatibility antigen-disparate) HCT by depleting T cells in case of a simultaneous CMV infection.

**Figure 10:**
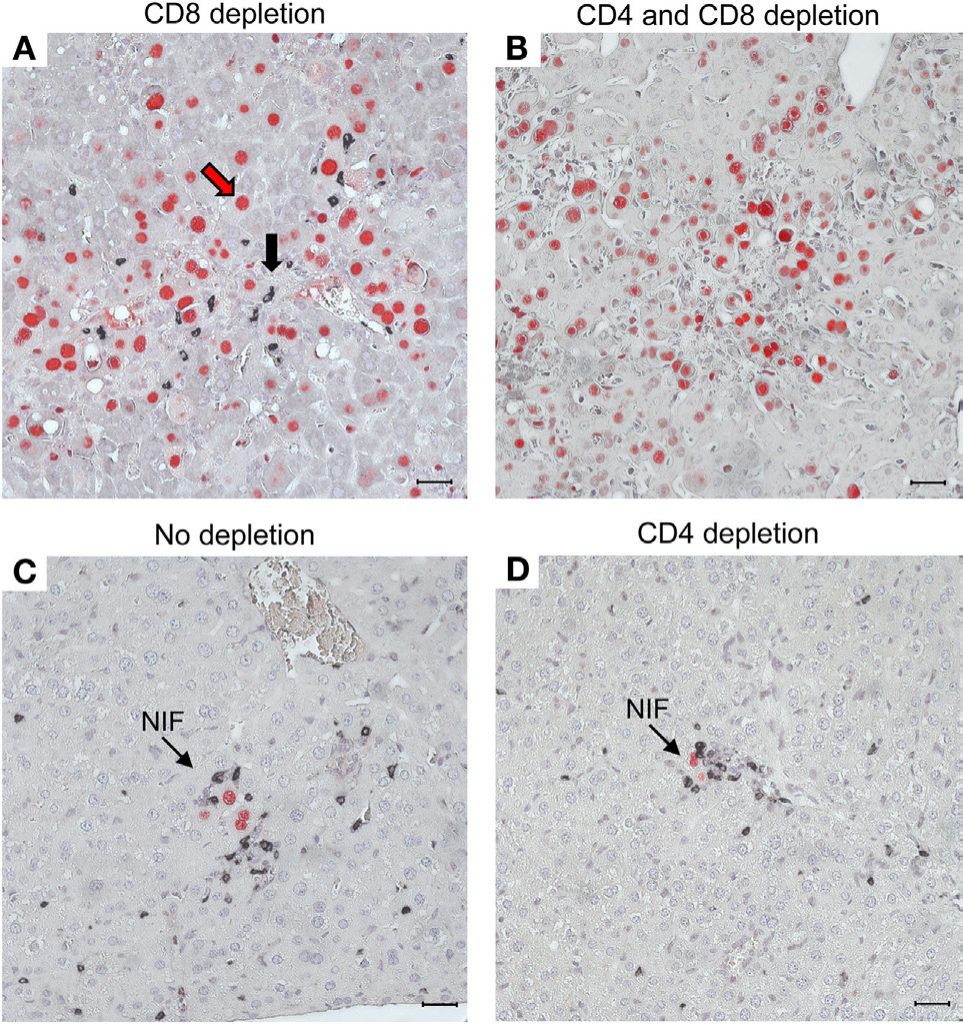
**CD8^+^ T cells are essential for confining the infection to nodular inflammatory foci (NIF) during reconstitution after HCT**. Two-color IHC images of viral pathology in the liver correspond to the survival/mortality data shown in Figure 9. **(A)** Uncontrolled virus spread in liver tissue after HCT and depletion of CD8^+^ T cells. The red arrow representatively points to an infected hepatocyte identified by red-staining of intranuclear IE1 protein. The black arrow representatively points to an infiltrating CD4^+^ T cell identified by black staining of cell surface CD3ε. Note that the CD4^+^ T cells are randomly distributed, do not form NIF, and apparently fail to limit the virus spread. **(B)** Lack of liver tissue infiltration, absence of NIF, and uncontrolled spread of the infection after depletion of both T-cell subsets, which excludes participation of other CD3ε-expressing immune cells, such as γ/δ T cells and NKT cells, in the control of infection. As the infection is not controlled, cells of innate immunity (unstained) apparently are also not functional in antiviral protection after HCT. **(C)** Confinement of infection to NIF in the absence of T-cell depletion. **(D)** Confinement of infection to NIF is maintained after depletion of CD4^+^ T cells, indicating that formation of NIF and control of the infection by CD8^+^ T cells do not critically require CD4^+^ T cell help. Bar markers represent 25 μm. Reproduced in rearranged form from Ref. (59) with permission from Caister Academic Press, Norfolk, UK.

As a histopathological correlate of mortality in the absence of CD8^+^ T cells, virus spreads uncontrolled in tissues, resulting in extended tissue damage [for the liver, see Figure 10; for the lungs and further organs, see Ref. (60, 61)]. Notably, the CD4^+^ T cells infiltrate infected liver tissue, but are distributed randomly, not forming protective NIF (Figure 10A). After depletion of both T-cell subsets, random and focal T-cell infiltrates are both missing and, accordingly, infection spreads uncontrolled (Figure 10B). As the IHC staining detected the CD3ε molecule of TCR–CD3 complexes, absence of stained infiltrates excluded a participation of γ/δ T cells as well as of NKT cells, both of which express CD3ε. Possibly present, though unstained, innate immune cells apparently failed to control the infection and were therefore not further considered. In contrast, in the absence of T-cell depletion, NIF are formed and infection is confined to few infected cells trapped in the center of NIF (Figure 10C). As NIF are also formed after depletion of CD4^+^ T cells (Figure 10D), CD8^+^ T cells obviously form the NIF and neither depend on CD4^+^ T-cell help nor on any other CD4^+^ cell type, for NIF formation and antiviral protection. As one may argue that CD8 depletion also depletes the CD8^+^ subset of DC, it is important to note that CD8^+^ T cells recovered from infected lungs and purified by cell sorting protected adoptive transfer recipients as a final proof of their direct antiviral effector function *in vivo* (61, 96).

In conclusion, in the HCT model of CMV infection, reconstitution of CD8^+^ T cells is the most critical parameter for the control of posttransplantation CMV infection. Thus, importantly, experimental HCT in the mouse model provided experimental “proof of principle” for the observational clinical evidence of CMV control by reconstituted CD8^+^ T cells. It should be emphasized that we have discussed here the resolution of acute organ infection required for preventing lethal CMV disease in HCT recipients. From a medical point of view, this immediate antiviral function is important for the patient in the first place. Notably, the above discussed HLA-transgenic mouse model of immunotherapy by adoptive cell transfer has revealed a benefit from adoptive cotransfer of TCR-transduced human CD4^+^ T cells in the control of antigenically “humanized” mCMV by TCR-transduced human CD8^+^ T cells (57). On the long run, CD4^+^ T cells reconstituted by HCT likely play a part in the long-term maintenance of protective CD8^+^ T cells and provide help for CD8^+^ T cell “memory inflation” during viral latency (118).

### A Novel Aspect in CMV Control: Mast Cells Enhance Protective Tissue Infiltration

Recent data in an immunocompetent mouse model have identified mast cells (MC) as a previously unconsidered player in the control of pulmonary CMV infection (116, 119, 120). The bottom-line message from these studies is that mCMV productively infects MC, causing their degranulation and release of the chemokine CCL5 (RANTES), which recruits antiviral CD8^+^ T cells to infected lungs where they transmigrate the capillary endothelium by diapedesis and form protective NIF within infected lung interstitium and parenchyma. Thus, in a negative feed-back loop, mCMV contributes to its own immune surveillance. Work in progress aims at demonstrating this function of MC also in the HCT model, and if so, a question will be whether enhanced CD8^+^ T-cell recruitment depends on radiation-resistant, tissue-resident MC of the recipient or on the reconstitution by donor MC derived from transplanted hematopoietic progenitors. It is postulated that enhanced recruitment of antiviral CD8^+^ T cells to infected lung tissue by MC-derived CCL5 compensates, at least in part, for the overall reduced hematopoietic reconstitution of CD8^+^ T cells and thereby contributes to the prevention, or at least moderation, of CMV pathology.

### Limited Importance of Viral Epitope Immunodominance for Control of Acute Infection

In any individual, only few viral CD8^+^ T-cell epitopes, that is virus-encoded antigenic peptides presented by host MHC-I molecules as peptide–MHC-I (pMHC-I) complexes, elicit a quantitatively dominant response. “Immunodominant epitopes” (IDEs) differ between human individuals (56) and between different mouse strains (30, 121), reflecting MHC-I polymorphism on the host population level. Immunodominance can, in theory, be determined by the primary “pre-immune” T-cell repertoire, including its spectrum of TCR affinity/avidity to pMHC-I and TCR expression density, by the efficiency of peptide processing and presentation in antigen-presenting cells (APCs), and by the extent of clonal expansion. Interestingly, the primary TCR repertoire can be variable even in genetically identical mice due to epigenetic differences, such as stochasticity in the TCR gene rearrangement [discussed and reviewed in Ref. (122–125)].

These parameters are linked in that clonal expansion depends on signaling intensity, which is defined not only by the affinity/avidity of TCR-pMHC-I interaction at the immunological synapse during the first contact with one professional APC but also on the probability of repeated subsequent antigen encounters with professional and/or non-professional APCs. Competition for growth factors, such as IL-2, is likely involved in clonal expansion and competition between clones. It is not an independent, T cell-intrinsic contributor, however, because clonotypic differences in IL-receptor expression levels cannot easily explain the epitope-specificity. Rather, epitope-specific enhanced signaling can upregulate the expression of IL receptors and thereby convey a growth advantage.

From these theoretical considerations one would have predicted that clones with high-avidity TCR–pMHC-I interactions gain a growth advantage, which would explain their “immunodominance” in quantity. Strikingly, the opposite appears to be the case. Relating the frequency of viral epitope-specific CD8^+^ T cells to their functional avidity, tested by an IFN-γ ELISpot assay with stimulator cells exogenously loaded with antigenic peptide at decreasing concentrations, response hierarchies to epitopes changed with the avidity threshold defined by peptide concentration. Specifically, at limiting peptide concentrations, the two most prominent IDEs of mCMV in BALB/c (H-2^d^) mice, namely m123/IE1 and m164, were no longer immunodominant (126). Thus, “immunodominance” reflects high numbers of low-avidity clones, whereas protection against infection is mediated by high-avidity clones (30, 127) capable of recognizing low numbers of pMHC-I complexes formed *in vivo* with limited amounts of naturally processed peptides. In addition, cell surface presentation of recently loaded pMHC-I complexes is limited by the action of viral immune evasion proteins that inhibit their cell surface trafficking [(128), reviewed in Ref. (129)].

It is conspicuous that IE genes are a coding hot spot for CD8^+^ T-cell immunogenicity of mCMV, confirmed more recently also for hCMV (see above). Interestingly, an immunodominant mCMV epitope, originally assigned to the early (E) phase protein m164/gp36.5 (130, 131), was recently shown to be also expressed in the IE phase from an upstream IE transcript (132). An explanation might be that the expression of IE genes, by definition, precedes the expression of downstream viral genes, including most of the viral immune evasion genes (18), thus providing a temporal advantage for T-cell priming with the consequence of IL consumption. In addition, cell type-dependent intrinsic host cell defense mechanisms and antiviral cytokines of the innate immune response might restrict *in vivo* gene expression in APCs to IE genes, and this could convey a selection advantage to CD8^+^ T cells specific for IE peptides. Sporadic episodes of IE gene expression during viral latency, associated with repetitive restimulation of cognate tissue-patrolling T celIs, drive “memory inflation” and thereby contribute to the high frequency of IE-specific CD8^+^ effector-memory T cells observed in latently infected tissues [reviewed in Ref. (48, 49)].

It is an underappreciated aspect that proteomic differences between virus strains also can determine the repertoire of IDEs by antigenicity-loss and antigenicity-gain mutations. In the BALB/c (H-2^d^) mouse model, four antigenic peptides (M105, m123/IE1, m145, and m164) have been classified as IDEs based on the response magnitude in the primary CD8^+^ T-cell response to acute mCMV infection (133). Notably, however, as shown by genetic deletion of all four of these IDEs, they are not essential for antiviral control in adoptive immunotherapy (133) and during reconstitution after HCT (117).

### Implications for a Preemptive Cytoimmunotherapy of CMV Infection after HCT

As T lymphopoietic reconstitution of antiviral CD8^+^ T cells following HCT takes time – too much time in cases of an early CMV reactivation – it makes sense to bridge the “window of risk” between HCT and reappearance of CD8^+^ T cells by adoptive transfer of virus-specific CD8^+^ effector or memory cells. Combining HCT with cytoimmunotherapy in the mouse model has indeed revealed a faster resolution of productive organ infection and an improved survival rate. In addition, long-term survivors of the combination therapy established latent infection with a lower viral genome load and, in consequence, a lower incidence of virus recurrence upon a second hemato-/immunoablation (134). Benefit from the combination therapy was confirmed in the meantime by clinical investigation (see above), except that, of course, the mouse model’s prediction of a reduced risk of secondary virus recurrence cannot be easily verified in patients.

As previous data in the mouse model have revealed a much higher protective efficacy of memory cells compared to effector cells of a CTLL with the very same viral epitope-specificity (114, 135), transfer of memory cells is preferable. Early data in the mouse model have revealed that an early intervention (preemptive cell transfer) is much more efficient than a later intervention when virus spread in tissues has already proceeded (therapeutic cell transfer) (26, 27). Prophylactic cell transfer with no preceding diagnosis of virus reactivation makes less sense, as virus reactivation is a stochastic event (136), which implies that the time of reactivation – and if it reactivates at all – is not predictable. Like with antiviral drugs, close PCR monitoring for CMV in the blood to start “preemptive” therapy upon first evidence for reactivation is currently the strategy of choice.

“Individualized medicine” in cytoimmunotherapy would require not only HLA typing, which is done anyway for HCT, but also knowledge of the epitopes encoded by the virus strain(s) harbored by latently infected CMV “seropositive” donors and recipients. As the reactivating CMV strain(s) are usually not known in advance and are rarely isolated and sequenced, CD8^+^ cytoimmunotherapy should not rely on a single viral epitope that might not be expressed by the reactivating virus, thereby causing a therapy failure. An alternative to be considered is a pretransplantation testing for the viral epitopes recognized by CD8^+^ T cells of donor and recipient. In principle, this is an option, since epitopes are known for the more common HLA types (137). Latent infection with multiple CMV strains poses a potential problem, because pretransplantation testing will reveal expression of a particular epitope by at least one of the strains, but not necessarily by the strain that reactivates. Thus, adoptive immunotherapy should not be clonal, but be polyclonal, with coverage of several epitopes presented by the respective set of HLA molecules.

A hitherto unconsidered argument for a preemptive CD8^+^ T-cell therapy is to avoid the viral pathogenesis not only in organs but also in BM stroma. Experiments in the murine model are in progress to evaluate this prospect.

## Concluding Simplified View of CMV Infection Control after HCT

**Figure 11:**
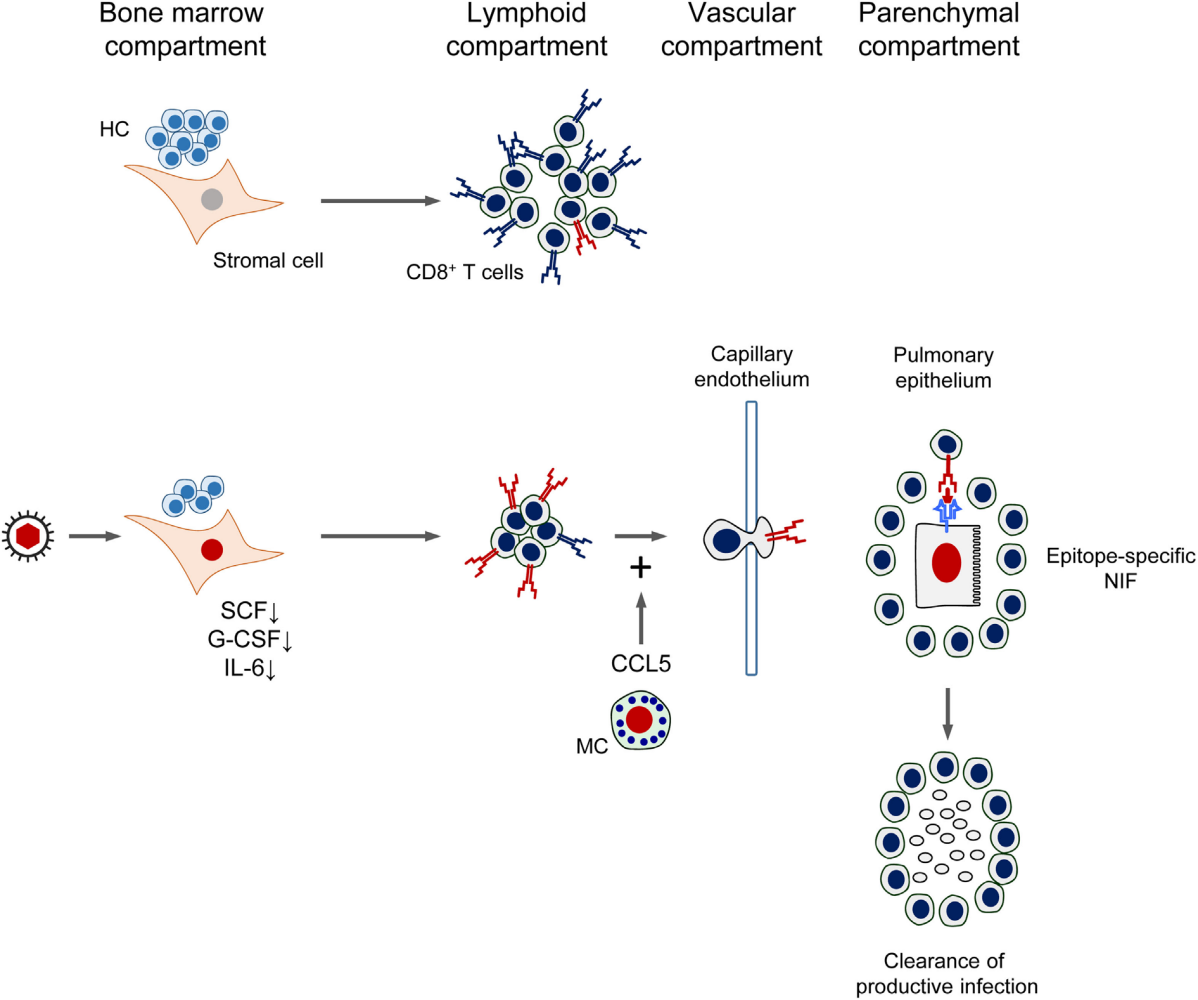
**Synopsis of the mutual interference between Cytomegalovirus and reconstitution of protective immunity after HCT**. See the body of the text for explanation. HC, hematopoietic cells; MC, mast cells; SCF, stem cell factor; G-CSF, granulocyte colony-stimulating factor; IL-6, interleukin-6; NIF, nodular inflammatory focus/foci. Red TCR symbol: CMV-specific. Blue TCR symbol: unrelated TCR specificity. Red-colored nuclei: infected cells.

As a synopsis, the mutual interference between CMV and CD8^+^ T-cell reconstitution after HCT is sketched in Figure 11. In essence, by infection of BM stromal cells, the expression of essential stroma-derived hemopoietins is reduced, which inhibits hematopoiesis. Reduced hematopoiesis in the BM entails a reduced T lymphopoiesis and eventually a reduced reconstitution of CD8^+^ T cells. This reduced reconstitution is in part functionally compensated by the infection of MC, which degranulate and release the chemokine CCL5 for recruiting the CD8^+^ T cells more efficiently to extralymphoid sites of infection. Thus recruited antiviral CD8^+^ T cells infiltrate infected tissue and migrate to infected tissue cells to form NIF in an epitope-specific manner. The CD8^+^ T cells in the NIF resolve productive infection by perforin-mediated cytolysis of the infected cells. After resolution of the productive infection, clusters of CD8^+^ T cells can be detected in tissue for some time as remnants of NIF.

## Author Contributions

The author confirms being the sole contributor of this work and approved it for publication.

## Conflict of Interest Statement

The author declares that the research was conducted in the absence of any commercial or financial relationships that could be construed as a potential conflict of interest.

## References

[1] Davison AJ Holton M Dolan A Dargan DJ Gatherer D Hayward GS Comparative genomics of primate cytomegaloviruses. In: Reddehase MJ, editor. Cytomegaloviruses: From Molecular Pathogenesis to Intervention. (Vol. 1), Norfolk, UK: Caister Academic Press (2013). p. 1–22.

[2] Ho M. The history of Cytomegalovirus and its diseases. Med Microbiol Immunol (2008) 197:65–73. 10.1007/s00430-007-0066-x 18087722

[3] Roizman B Sears AE An inquiry into the mechanisms of herpes simplex virus latency. Annu Rev Microbiol (1987) 41:543–71. 10.1146/annurev.mi.41.100187.002551 2825588

[4] Poole E Sinclair J. Sleepless latency of human Cytomegalovirus. Med Microbiol Immunol (2015) 204:421–9. 10.1007/s00430-015-0401-6 25772624 PMC4439429

[5] Reeves M Sinclair J Epigenetic regulation of human Cytomegalovirus gene expression: impact on latency and reactivation. In: Reddehase MJ, editor. Cytomegaloviruses: From Molecular Pathogenesis to Intervention. (Vol. 1), Norfolk, UK: Caister Academic Press (2013). p. 330–46.

[6] Slobedman B Avdic S Abendroth A Transcription associated with human Cytomegalovirus latency. In: Reddehase MJ, editor. Cytomegaloviruses: From Molecular Pathogenesis to Intervention. (Vol. 1), Norfolk, UK: Caister Academic Press (2013). p. 347–62.

[7] Cannon MJ Grosse SD Fowler KB The epidemiology and public health impact of congenital Cytomegalovirus infection. In: Reddehase MJ, editor. Cytomegaloviruses: From Molecular Pathogenesis to Intervention. (Vol. 2), Norfolk, UK: Caister Academic Press (2013). p. 26–48.

[8] Adler SP Nigro G Clinical Cytomegalovirus research: congenital infection. In: Reddehase MJ, editor. Cytomegaloviruses: From Molecular Pathogenesis to Intervention. (Vol. 2), Norfolk, UK: Caister Academic Press (2013). p. 55–72.

[9] Hummel M Abecassis MM. A model for reactivation of CMV from latency. J Clin Virol (2002) 25:S123–36. 10.1016/S1386-6532(02)00088-4 12361763

[10] Kim SJ Varghese TK Zhang Z Zhao LC Thomas G Hummel M Renal ischemia/reperfusion injury activates the enhancer domain of the human Cytomegalovirus major immediate early promoter. Am J Transplant (2005) 5:1606–13. 10.1111/j.1600-6143.2005.00912.x 15943618

[11] Boppana SB Britt WJ Synopsis of clinical aspects of human Cytomegalovirus disease. In: Reddehase MJ, editor. Cytomegaloviruses: From Molecular Pathogenesis to Intervention. (Vol. 2), Norfolk, UK: Caister Academic Press (2013). p. 1–25.

[12] Sissons JG Wills MR. How understanding immunology contributes to managing CMV disease in immunosuppressed patients: now and in future. Med Microbiol Immunol (2015) 204:307–16. 10.1007/s00430-015-0415-0 25896527

[13] Smith SM Streblow DN Caposio P Nelson JA Humanized mouse models of Cytomegalovirus pathogenesis and latency. In: Reddehase MJ, editor. Cytomegaloviruses: From Molecular Pathogenesis to Intervention. (Vol. 1), Norfolk, UK: Caister Academic Press (2013). p. 417–36.

[14] Smith MG Propagation in tissue cultures of a cytopathogenic virus from human salivary gland virus (SGV) disease. Proc Soc Exp Biol Med (1956) 92:424–30. 10.3181/00379727-92-22498 13350368

[15] Reddehase MJ Margaret Gladys Smith, mother of Cytomegalovirus: 60th anniversary of Cytomegalovirus isolation. Med Microbiol Immunol (2015) 204:239–41. 10.1007/s00430-015-0416-z 25864083

[16] Ostermann E Pawletko K Indenbirken D Schumacher U Brune W. Stepwise adaptation of murine Cytomegalovirus to cells of a foreign host for identification of host range determinants. Med Microbiol Immunol (2015) 204:461–9. 10.1007/s00430-015-0400-7 25788395

[17] Redwood AJ Shellam GR Smith LM Molecular evolution of murine Cytomegalovirus genomes. In: Reddehase MJ, editor. Cytomegaloviruses: From Molecular Pathogenesis to Intervention. (Vol. 1), Norfolk, UK: Caister Academic Press (2013). p. 23–37.

[18] Reddehase MJ. Antigens and immunoevasins: opponents in Cytomegalovirus immune surveillance. Nat Rev Immunol (2002) 2:831–44. 10.1038/nri932 12415307

[19] Powers C DeFilippis V Malouli D Früh K. Cytomegalovirus immune evasion. Curr Top Microbiol Immunol (2008) 325:333–59. 10.1007/978-3-540-77349-8_19 18637515

[20] Vidal S Krmpotic A Pyzik M Jonjic S Innate immunity to Cytomegalovirus in the murine model. In: Reddehase MJ, editor. Cytomegaloviruses: From Molecular Pathogenesis to Intervention. (Vol. 2), Norfolk, UK: Caister Academic Press (2013). p. 191–213.

[21] Mc Gregor A McVoy MA Schleiss MR The guinea pig model of congenital Cytomegalovirus infection. In: Reddehase MJ, editor. Cytomegaloviruses: From Molecular Pathogenesis to Intervention. (Vol. 2), Norfolk, UK: Caister Academic Press (2013). p. 87–117.

[22] Voigt S Ettinger J Streblow DN The rat model of Cytomegalovirus infection and vascular disease. In: Reddehase MJ, editor. Cytomegaloviruses: From Molecular Pathogenesis to Intervention. (Vol. 2), Norfolk, UK: Caister Academic Press (2013). p. 310–34.

[23] Früh K Malouli D Oxford KL Barry PA Non-human-primate models of Cytomegalovirus infection, prevention, and therapy. In: Reddehase MJ, editor. Cytomegaloviruses: From Molecular Pathogenesis to Intervention. (Vol. 2), Norfolk, UK: Caister Academic Press (2013). p. 461–94.

[24] Benedict CA Crozat K Degli-Esposti M Dalod M Host genetic models in Cytomegalovirus immunology. In: Reddehase MJ, editor. Cytomegaloviruses: From Molecular Pathogenesis to Intervention. (Vol. 2), Norfolk, UK: Caister Academic Press (2013). p. 310–34.

[25] Wilkinson GW Davison AJ Tomasec P Fielding CA Aicheler R Murrell I Human Cytomegalovirus: taking the strain. Med Microbiol Immunol (2015) 204:273–84. 10.1007/s00430-015-0411-4 25894764 PMC4439430

[26] Reddehase MJ Weiland F Münch K Jonjic S Lüske A Koszinowski UH. Interstitial murine Cytomegalovirus pneumonia after irradiation: characterization of cells that limit viral replication during established infection of the lungs. J Virol (1985) 55:264–73. 2991554 10.1128/jvi.55.2.264-273.1985 PMC254929

[27] Reddehase MJ Mutter W Koszinowski UH. In vivo application of recombinant interleukin 2 in the immunotherapy of established Cytomegalovirus infection. J Exp Med (1987) 165:650–6. 10.1084/jem.165.3.650 3029272 PMC2188275

[28] Reddehase MJ Jonjić S Weiland F Mutter W Koszinowski UH. Adoptive immunotherapy of murine Cytomegalovirus adrenalitis in the immunocompromised host: CD4-helper-independent antiviral function of CD8-positive memory T lymphocytes derived from latently infected donors. J Virol (1988) 62:1061–5. 2828654 10.1128/jvi.62.3.1061-1065.1988 PMC253668

[29] Holtappels R Böhm V Podlech J Reddehase MJ. CD8 T-cell-based immunotherapy of Cytomegalovirus infection: “proof of concept” provided by the murine model. Med Microbiol Immunol (2008) 197:125–34. 10.1007/s00430-008-0093-2 18343947

[30] Ebert S Podlech J Gillert-Marien D Gergely KM Büttner JK Fink A Parameters determining the efficacy of adoptive CD8 T-cell therapy of Cytomegalovirus infection. Med Microbiol Immunol (2012) 201:527–39. 10.1007/s00430-012-0258-x 22972232

[31] Riddell SR Watanabe KS Goodrich JM Li CR Agha ME Greenberg PD. Restoration of viral immunity in immunodeficient humans by the adoptive transfer of T cell clones. Science (1992) 257:238–41. 10.1126/science.1352912 1352912

[32] Walter EA Greenberg PD Gilbert MJ Finch RJ Watanabe KS Thomas ED Reconstitution of cellular immunity against Cytomegalovirus in recipients of allogeneic bone marrow by transfer of T-cell clones from the donor. N Engl J Med (1995) 333:1038–44. 10.1056/NEJM199510193331603 7675046

[33] Einsele H Roosnek E Rufer N Sinzger C Riegler S Löffler J Infusion of Cytomegalovirus (CMV)-specific T cells for the treatment of CMV infection not responding to antiviral chemotherapy. Blood (2002) 99:3916–22. 10.1182/blood.V99.11.3916 12010789

[34] Peggs KS Verfuerth S Pizzey A Khan N Guiver M Moss PA Adoptive cellular therapy for early Cytomegalovirus infection after allogeneic stem-cell transplantation with virus-specific T-cell lines. Lancet (2003) 362:1375–7. 10.1016/S0140-6736(03)14634-X 14585640

[35] Cobbold M Khan N Pourgheysari B Tauro S McDonald D Osman H Adoptive transfer of Cytomegalovirus-specific CTL to stem cell transplant patients after selection by HLA-peptide tetramers. J Exp Med (2005) 202:379–86. 10.1084/jem.20040613 16061727 PMC2213070

[36] Odendahl M Grigoleit GU Bönig H Neuenhahn M Albrecht J Anderl F Clinical-scale isolation of ‘minimally manipulated’ Cytomegalovirus-specific donor lymphocytes for the treatment of refractory Cytomegalovirus disease. Cytotherapy (2014) 16:1245–56. 10.1016/j.jcyt.2014.05.023 25108651

[37] Reddehase MJ Koszinowski UH. Significance of herpesvirus immediate early gene expression in cellular immunity to Cytomegalovirus infection. Nature (1984) 312:369–71. 10.1038/312369a0 6095095

[38] Reddehase MJ Mutter W Münch K Bühring HJ Koszinowski UH. CD8-positive T lymphocytes specific for murine Cytomegalovirus immediate-early antigens mediate protective immunity. J Virol (1987) 61:3102–8. 3041033 10.1128/jvi.61.10.3102-3108.1987 PMC255886

[39] Reddehase MJ Rothbard JB Koszinowski UH. A pentapeptide as minimal antigenic determinant for MHC class I-restricted T lymphocytes. Nature (1989) 337:651–3. 10.1038/337651a0 2465495

[40] Del Val M Schlicht HJ Volkmer H Messerle M Reddehase MJ Koszinowski UH. Protection against lethal Cytomegalovirus infection by a recombinant vaccine containing a single nonameric T-cell epitope. J Virol (1991) 65:3641–6. 1710286 10.1128/jvi.65.7.3641-3646.1991 PMC241372

[41] Stinski MF Isomura H. Role of the Cytomegalovirus major immediate early enhancer in acute infection and reactivation from latency. Med Microbiol Immunol (2008) 197:223–31. 10.1007/s00430-007-0069-7 18097687

[42] Kurz SK Rapp M Steffens HP Grzimek NK Schmalz S Reddehase MJ. Focal transcriptional activity of murine Cytomegalovirus during latency in the lungs. J Virol (1999) 73:482–94. 9847354 10.1128/jvi.73.1.482-494.1999 PMC103855

[43] Grzimek NK Dreis D Schmalz S Reddehase MJ. Random, asynchronous, and asymmetric transcriptional activity of enhancer-flanking major immediate-early genes ie1/3 and ie2 during murine Cytomegalovirus latency in the lungs. J Virol (2001) 75:2692–705. 10.1128/JVI.75.6.2692-2705.2001 11222693 PMC115894

[44] Simon CO Holtappels R Tervo HM Böhm V Däubner T Oehrlein-Karpi SA CD8 T cells control Cytomegalovirus latency by epitope-specific sensing of transcriptional reactivation. J Virol (2006) 80:10436–56. 10.1128/JVI.01248-06 16928768 PMC1641801

[45] Reddehase MJ Simon CO Seckert CK Lemmermann N Grzimek NK. Murine model of Cytomegalovirus latency and reactivation. Curr Top Microbiol Immunol (2008) 325:315–31. 10.1007/978-3-540-77349-8_18 18637514

[46] Holtappels R Pahl-Seibert MF Thomas D Reddehase MJ. Enrichment of immediate-early 1 (m123/pp89) peptide-specific CD8 T cells in a pulmonary CD62L(lo) memory-effector cell pool during latent murine Cytomegalovirus infection of the lungs. J Virol (2000) 74:11495–503. 10.1128/JVI.74.24.11495-11503.2000 11090146 PMC112429

[47] Karrer U Sierro S Wagner M Oxenius A Hengel H Koszinowski UH Memory inflation: continuous accumulation of antiviral CD8+ T cells over time. J Immunol (2003) 170:2022–9; Correction in *J Immunol* (2003) 17:3895. 10.4049/jimmunol.170.4.2022 12574372

[48] Seckert CK Griessl M Büttner JK Scheller S Simon CO Kropp KA Viral latency drives ‘memory inflation’: a unifying hypothesis linking two hallmarks of Cytomegalovirus infection. Med Microbiol Immunol (2012) 201:551–66. 10.1007/s00430-012-0273-y 22991040

[49] Seckert CK Grießl M Büttner JK Freitag K Lemmermann NA Hummel MA Immune surveillance of Cytomegalovirus latency and reactivation in murine models: link to ‘memory inflation’. In: Reddehase MJ, editor. Cytomegaloviruses: From Molecular Pathogenesis to Intervention. (Vol. 1), Norfolk, UK: Caister Academic Press (2013). p. 374–416.

[50] Borysiewicz LK Hickling JK Graham S Sinclair J Cranage MP Smith GL Human Cytomegalovirus-specific cytotoxic T cells. Relative frequency of stage-specific CTL recognizing the 72-kD immediate early protein and glycoprotein B expressed by recombinant vaccinia viruses. J Exp Med (1988) 168:919–31. 10.1084/jem.168.3.919 2844952 PMC2189029

[51] McLaughlin-Taylor E Pande H Forman SJ Tanamachi B Li CR Zaia JA Identification of the major late human Cytomegalovirus matrix protein pp65 as a target antigen for CD8+ virus-specific cytotoxic T lymphocytes. J Med Virol (1994) 43:103–10. 10.1002/jmv.1890430119 8083644

[52] Wills MR Carmichael AJ Mynard K Jin X Weekes MP Plachter B The human cytotoxic T-lymphocyte (CTL) response to Cytomegalovirus is dominated by structural protein pp65: frequency, specificity, and T-cell receptor usage of pp65-specific CTL. J Virol (1996) 70:7569–79. 8892876 10.1128/jvi.70.11.7569-7579.1996 PMC190825

[53] Büscher N Paulus C Nevels M Tenzer S Plachter B. The proteome of human Cytomegalovirus virions and dense bodies is conserved across different strains. Med Microbiol Immunol (2015) 204:285–93. 10.1007/s00430-015-0397-y 25732096

[54] Kern F Surel IP Faulhaber N Frömmel C Schneider-Mergener J Schönemann C Target structures of the CD8(+)-T-cell response to human Cytomegalovirus: the 72-kilodalton major immediate-early protein revisited. J Virol (1999) 73:8179–84. 10482568 10.1128/jvi.73.10.8179-8184.1999 PMC112835

[55] Bunde T Kirchner A Hoffmeister B Habedank D Hetzer R Cherepnev G Protection from Cytomegalovirus after transplantation is correlated with immediate early 1-specific CD8 T cells. J Exp Med (2005) 201:1031–6. 10.1084/jem.20042384 15795239 PMC2213133

[56] Sylwester AW Mitchell BL Edgar JB Taormina C Pelte C Ruchti F Broadly targeted human Cytomegalovirus-specific CD4+ and CD8+ T cells dominate the memory compartments of exposed subjects. J Exp Med (2005) 202:673–85. 10.1084/jem.20050882 16147978 PMC2212883

[57] Thomas S Klobuch S Podlech J Plachter B Hoffmann P Renzaho A Evaluating human T-cell therapy of Cytomegalovirus organ disease in HLA-transgenic mice. PLoS Pathog (2015) 11:e1005049. 10.1371/journal.ppat.1005049 26181057 PMC4504510

[58] Revello MG Gerna G State of the art and trends in Cytomegalovirus diagnostics. In: Reddehase MJ, editor. Cytomegaloviruses: From Molecular Pathogenesis to Intervention. (Vol. 2), Norfolk, UK: Caister Academic Press (2013). p. 380–99.

[59] Holtappels R Ebert S Podlech J Fink A Böhm V Lemmermann NA Murine model for cytoimmunotherapy of CMV disease after haematopoietic cell transplantation. In: Reddehase MJ, editor. Cytomegaloviruses: From Molecular Pathogenesis to Intervention. (Vol. 2), Norfolk, UK: Caister Academic Press (2013). p. 352–79.

[60] Podlech J Holtappels R Wirtz N Steffens HP Reddehase MJ. Reconstitution of CD8 T cells is essential for the prevention of multiple-organ Cytomegalovirus histopathology after bone marrow transplantation. J Gen Virol (1998) 79:2099–104. 10.1099/0022-1317-79-9-2099 9747717

[61] Podlech J Holtappels R Pahl-Seibert MF Steffens HP Reddehase MJ. Murine model of interstitial Cytomegalovirus pneumonia in syngeneic bone marrow transplantation: persistence of protective pulmonary CD8-T-cell infiltrates after clearance of acute infection. J Virol (2000) 74:7496–507. 10.1128/JVI.74.16.7496-7507.2000 10906203 PMC112270

[62] Bianco P Riminucci M Gronthos S Robey PG. Bone marrow stromal stem cells: nature, biology, and potential applications. Stem Cells (2001) 19:180–92. 10.1634/stemcells.19-3-180 11359943

[63] Busch FW Mutter W Koszinowski UH Reddehase MJ. Rescue of myeloid lineage-committed preprogenitor cells from Cytomegalovirus-infected bone marrow stroma. J Virol (1991) 65:981–4. 1846211 10.1128/jvi.65.2.981-984.1991 PMC239843

[64] Apperley JF Dowding C Hibbin J Buiter J Matutes E Sissons PJ The effect of Cytomegalovirus on hemopoiesis: in vitro evidence for selective infection of marrow stromal cells. Exp Hematol (1989) 17:38–45. 2535697

[65] Simmons P Kaushansky K Torok-Storb B. Mechanisms of Cytomegalovirus-mediated myelosuppression: perturbation of stromal cell function versus direct infection of myeloid cells. Proc Natl Acad Sci U S A (1990) 87:1386–90. 10.1073/pnas.87.4.1386 2154745 PMC53480

[66] Taylor-Wiedeman J Sissons JG Borysiewicz LK Sinclair JH. Monocytes are a major site of persistence of human Cytomegalovirus in peripheral blood mononuclear cells. J Gen Virol (1991) 72:2059–64. 10.1099/0022-1317-72-9-2059 1654370

[67] Maciejewski JP Bruening EE Donahue RE Mocarski ES Young NS St Jeor SC. Infection of hematopoietic progenitor cells by human Cytomegalovirus. Blood (1992) 80:170–8. 1377049

[68] Söderberg-Nauclér C Fish KN Nelson JA. Reactivation of latent human Cytomegalovirus by allogeneic stimulation of blood cells from healthy donors. Cell (1997) 91:119–26. 10.1016/S0092-8674(01)80014-3 9335340

[69] Hahn G Jores R Mocarski ES. Cytomegalovirus remains latent in a common precursor of dendritic and myeloid cells. Proc Natl Acad Sci U S A (1998) 95:3937–42. 10.1073/pnas.95.7.3937 9520471 PMC19941

[70] Goodrum FD Jordan CT High K Shenk T. Human Cytomegalovirus gene expression during infection of primary hematopoietic progenitor cells: a model for latency. Proc Natl Acad Sci U S A (2002) 99:16255–60. 10.1073/pnas.252630899 12456880 PMC138598

[71] Emery VC Relative importance of Cytomegalovirus load as a risk factor for Cytomegalovirus disease in the immunocompromised host. In: Scholz M Rabenau HF Doerr HW Cinatl J, editors. CMV-Related Immunopathology. Basel, Swiss: Karger (1998). p. 288–301.

[72] Crapnell KB Almeida-Porada G Khaiboullina S St Jeor SC Zanjani ED. Human haematopoietic stem cells that mediate long-term in vivo engraftment are not susceptible to infection by human Cytomegalovirus. Br J Haematol (2004) 124:676–84. 10.1111/j.1365-2141.2004.04827.x 14871256

[73] Seckert CK Renzaho A Reddehase MJ Grzimek NK. Hematopoietic stem cell transplantation with latently infected donors does not transmit virus to immunocompromised recipients in the murine model of Cytomegalovirus infection. Med Microbiol Immunol (2008) 197:251–9. 10.1007/s00430-008-0094-1 18365252

[74] Adler B Sinzger C Cytomegalovirus interstrain variance in cell type tropism. In: Reddehase MJ, editor. Cytomegaloviruses: From Molecular Pathogenesis to Intervention. (Vol. 1), Norfolk, UK: Caister Academic Press (2013). p. 297–321.

[75] Wagner FM Brizic I Prager A Trsan T Arapovic M Lemmermann NA The viral chemokine MCK-2 of murine Cytomegalovirus promotes infection as part of a gH/gL/MCK-2 complex. PLoS Pathog (2013) 9:e1003493. 10.1371/journal.ppat.1003493 23935483 PMC3723581

[76] Lemmermann NA Krmpotic A Podlech J Brizic I Prager A Adler H Non-redundant and redundant roles of Cytomegalovirus gH/gL complexes in host organ entry and intra-tissue spread. PLoS Pathog (2015) 11:e1004640. 10.1371/journal.ppat.1004640 25659098 PMC4450070

[77] Messerle M Crnkovic I Hammerschmidt W Ziegler H Koszinowski UH. Cloning and mutagenesis of a herpesvirus genome as an infectious bacterial artificial chromosome. Proc Natl Acad Sci U S A (1997) 94:14759–63. 10.1073/pnas.94.26.14759 9405686 PMC25110

[78] Wagner M Jonjic S Koszinowski UH Messerle M. Systematic excision of vector sequences from the BAC-cloned herpesvirus genome during virus reconstitution. J Virol (1999) 73:7056–60. 10400809 10.1128/jvi.73.8.7056-7060.1999 PMC112796

[79] Lemmermann NA Kropp KA Seckert CK Grzimek NK Reddehase MJ. Reverse genetics modification of Cytomegalovirus antigenicity and immunogenicity by CD8 T-cell epitope deletion and insertion. J Biomed Biotechnol (2011) 2011:812742. 10.1155/2011/812742 21253509 PMC3021883

[80] Jordan S Krause J Prager A Mitrovic M Jonjic S Koszinowski UH Virus progeny of murine Cytomegalovirus bacterial artificial chromosome pSM3fr show reduced growth in salivary glands due to a fixed mutation of MCK-2. J Virol (2011) 85:10346–53. 10.1128/JVI.00545-11 21813614 PMC3196435

[81] Mutter W Reddehase MJ Busch FW Bühring HJ Koszinowski UH. Failure in generating hemopoietic stem cells is the primary cause of death from Cytomegalovirus disease in the immunocompromised host. J Exp Med (1988) 167:1645–58. 10.1084/jem.167.5.1645 2896757 PMC2188951

[82] Reddehase MJ Bone marrow dysfunction in irradiated, Cytomegalovirus-infected mice. Transplant Proc (1991) 23:10–1. 1648809

[83] Reddehase MJ Dreher-Stumpp L Angele P Balthesen M Susa M. Hematopoietic stem cell deficiency resulting from Cytomegalovirus infection of bone marrow stroma. Ann Hematol (1992) 64:A125–7. 10.1007/BF01715364 1322181

[84] Mayer A Podlech J Kurz S Steffens HP Maiberger S Thalmeier K Bone marrow failure by Cytomegalovirus is associated with an in vivo deficiency in the expression of essential stromal hemopoietin genes. J Virol (1997) 71:4589–98. 9151853 10.1128/jvi.71.6.4589-4598.1997 PMC191681

[85] Dobonici M Podlech J Steffens HP Maiberger S Reddehase MJ. Evidence against a key role for transforming growth factor-beta1 in Cytomegalovirus-induced bone marrow aplasia. J Gen Virol (1998) 79:867–76. 10.1099/0022-1317-79-4-867 9568983

[86] Steffens HP Podlech J Kurz S Angele P Dreis D Reddehase MJ. Cytomegalovirus inhibits the engraftment of donor bone marrow cells by downregulation of hemopoietin gene expression in recipient stroma. J Virol (1998) 72:5006–15. 9573270 10.1128/jvi.72.6.5006-5015.1998 PMC110063

[87] Chabannon C Torok-Storb B Stem cell-stromal cell interactions. Curr Top Microbiol Immunol (1992) 177:123–36. 1379138 10.1007/978-3-642-76912-2_10

[88] Schofield R The pluripotent stem cell. Clin Haematol (1979) 8:221–37. 226304

[89] Heazlewood SY Oteiza A Cao H Nilsson SK. Analyzing hematopoietic stem cell homing, lodgment, and engraftment to better understand the bone marrow niche. Ann N Y Acad Sci (2014) 1310:119–28. 10.1111/nyas.12329 24428368

[90] Birbrair A Frenette PS Niche heterogeneity in the bone marrow. Ann N Y Acad Sci (2016) 1370:82–96. 10.1111/nyas.13016 27015419 PMC4938003

[91] Hoggatt J Kfoury Y Scadden DT. Hematopoietic stem cell niche in health and disease. Annu Rev Pathol (2016) 11:555–81. 10.1146/annurev-pathol-012615-044414 27193455

[92] Yu VW Scadden DT. Heterogeneity of the bone marrow niche. Curr Opin Hematol (2016) 23:331–8. 10.1097/MOH.0000000000000265 27177311 PMC6819995

[93] Driessen RL Johnston HM Nilsson SK. Membrane-bound stem cell factor is a key regulator in the initial lodgment of stem cells within the endosteal marrow region. Exp Hematol (2003) 31:1284–91. 10.1016/j.exphem.2003.08.015 14662336

[94] Quinnan GV Kirmani N Rook AH Manischewitz JF Jackson L Moreschi G Cytotoxic T cells in Cytomegalovirus infection: HLA-restricted T-lymphocyte and non-T-lymphocyte cytotoxic responses correlate with recovery from Cytomegalovirus infection in bone-marrow-transplant recipients. N Engl J Med (1982) 307:7–13. 10.1056/NEJM198207013070102 6281647

[95] Reusser P Riddell SR Meyers JD Greenberg PD. Cytotoxic T-lymphocyte response to Cytomegalovirus after human allogeneic bone marrow transplantation: pattern of recovery and correlation with Cytomegalovirus infection and disease. Blood (1991) 78:1373–80. 1652311

[96] Alterio de Goss M Holtappels R Steffens HP Podlech J Angele P Dreher L Control of Cytomegalovirus in bone marrow transplantation chimeras lacking the prevailing antigen-presenting molecule in recipient tissues rests primarily on recipient-derived CD8 T cells. J Virol (1998) 72:7733–44. 9733809 10.1128/jvi.72.10.7733-7744.1998 PMC110079

[97] Jonjić S Pavić I Lucin P Rukavina D Koszinowski UH. Efficacious control of Cytomegalovirus infection after long-term depletion of CD8+ T lymphocytes. J Virol (1990) 64:5457–64. 1976821 10.1128/jvi.64.11.5457-5464.1990 PMC248597

[98] Polić B Jonjić S Pavić I Crnković I Zorica I Hengel H Lack of MHC class I complex expression has no effect on spread and control of Cytomegalovirus infection in vivo. J Gen Virol (1996) 77:217–25. 10.1099/0022-1317-77-2-217 8627225

[99] Khairallah C Netzer S Villacreces A Juzan M Rousseau B Dulanto S γδ T cells confer protection against murine Cytomegalovirus (MCMV). PLoS Pathog (2015) 11:e1004702. 10.1371/journal.ppat.1004702 25747674 PMC4352080

[100] Bukowski JF Warner JF Dennert G Welsh RM. Adoptive transfer studies demonstrating the antiviral effect of natural killer cells in vivo. J Exp Med (1985) 161:40–52. 10.1084/jem.161.1.40 2981954 PMC2187554

[101] Klenovsek K Weisel F Schneider A Appelt U Jonjic S Messerle M Protection from CMV infection in immunodeficient hosts by adoptive transfer of memory B cells. Blood (2007) 110:3472–9. 10.1182/blood-2007-06-095414 17656648

[102] Jeitziner SM Walton SM Torti N Oxenius A. Adoptive transfer of Cytomegalovirus-specific effector CD4+ T cells provides antiviral protection from murine CMV infection. Eur J Immunol (2013) 43:2886–95. 10.1002/eji.201343690 23921569

[103] Sell S Dietz M Schneider A Holtappels R Mach M Winkler TH. Control of murine Cytomegalovirus infection by γδ T cells. PLoS Pathog (2015) 11:e1004481. 10.1371/journal.ppat.1004481 25658831 PMC4450058

[104] Riddell SR. Pathogenesis of Cytomegalovirus pneumonia in immunocompromised hosts. Semin Respir Infect (1995) 10:199–208. 8668847

[105] Seo S Boeckh M Clinical Cytomegalovirus research: haematopoietic cell transplantation. In: Reddehase MJ, editor. Cytomegaloviruses: From Molecular Pathogenesis to Intervention. (Vol. 2), Norfolk, UK: Caister Academic Press (2013). p. 335–51.

[106] Brody AR Craighead JE Pathogenesis of pulmonary Cytomegalovirus infection in immunosuppressed mice. J Infect Dis (1974) 129:677–89. 10.1093/infdis/129.6.677 4365945

[107] Jordan MC. Interstitial pneumonia and subclinical infection after intranasal inoculation of murine Cytomegalovirus. Infect Immun (1978) 21:275–80. 213384 10.1128/iai.21.1.275-280.1978 PMC421986

[108] Shanley JD Pesanti EL Nugent KM. The pathogenesis of pneumonitis due to murine Cytomegalovirus. J Infect Dis (1982) 146:388–96. 10.1093/infdis/146.3.388 6286796

[109] Holtappels R Podlech J Geginat G Steffens HP Thomas D Reddehase MJ Control of murine Cytomegalovirus in the lungs: relative but not absolute immunodominance of the immediate-early 1 nonapeptide during the antiviral cytolytic T-lymphocyte response in pulmonary infiltrates. J Virol (1998) 72:7201–12. 9696814 10.1128/jvi.72.9.7201-7212.1998 PMC109942

[110] Balthesen M Messerle M Reddehase MJ. Lungs are a major organ site of Cytomegalovirus latency and recurrence. J Virol (1993) 67:5360–6. 8394453 10.1128/jvi.67.9.5360-5366.1993 PMC237936

[111] Kataoka N Shinohara H Takayama K Takaku K Kondo S Yonehara S Concanamycin A, a powerful tool for characterization and estimation of contribution of perforin- and Fas-based lytic pathways in cell-mediated cytotoxicity. J Immunol (1996) 156:3678–86. 8621902

[112] Seckert CK Schader SI Ebert S Thomas D Freitag K Renzaho A Antigen-presenting cells of haematopoietic origin prime Cytomegalovirus-specific CD8 T-cells but are not sufficient for driving memory inflation during viral latency. J Gen Virol (2011) 92:1994–2005. 10.1099/vir.0.031815-0 21632567

[113] Lefrançois L Obar JJ. Once a killer, always a killer: from cytotoxic T cell to memory cell. Immunol Rev (2010) 235:206–18. 10.1111/j.0105-2896.2010.00895.x 20536565 PMC2989239

[114] Böhm V Podlech J Thomas D Deegen P Pahl-Seibert MF Lemmermann NA Epitope-specific in vivo protection against Cytomegalovirus disease by CD8 T cells in the murine model of preemptive immunotherapy. Med Microbiol Immunol (2008) 197:135–44. 10.1007/s00430-008-0092-3 18340461

[115] Sacher T Podlech J Mohr CA Jordan S Ruzsics Z Reddehase MJ The major virus-producing cell type during murine Cytomegalovirus infection, the hepatocyte, is not the source of virus dissemination in the host. Cell Host Microbe (2008) 3:263–72. 10.1016/j.chom.2008.02.014 18407069

[116] Ebert S Becker M Lemmermann NA Büttner JK Michel A Taube C Mast cells expedite control of pulmonary murine Cytomegalovirus infection by enhancing the recruitment of protective CD8 T cells to the lungs. PLoS Pathog (2014) 10:e1004100. 10.1371/journal.ppat.1004100 24763809 PMC3999167

[117] Holtappels R Lemmermann NA Podlech J Ebert S Reddehase MJ. Reconstitution of CD8 T cells protective against Cytomegalovirus in a mouse model of hematopoietic cell transplantation: dynamics and inessentiality of epitope immunodominance. Front Immunol (2016) 7:232. 10.3389/fimmu.2016.00232 27379095 PMC4905951

[118] Walton SM Torti N Mandaric S Oxenius A. T-cell help permits memory CD8(+) T-cell inflation during Cytomegalovirus latency. Eur J Immunol (2011) 41:2248–59. 10.1002/eji.201141575 21590767

[119] Becker M Lemmermann NA Ebert S Baars P Renzaho A Podlech J Mast cells as rapid innate sensors of Cytomegalovirus by TLR3/TRIF signaling-dependent and -independent mechanisms. Cell Mol Immunol (2015) 12:192–201. 10.1038/cmi.2014.73 25152077 PMC4654297

[120] Podlech J Ebert S Becker M Reddehase MJ Stassen M Lemmermann NA. Mast cells: innate attractors recruiting protective CD8 T cells to sites of Cytomegalovirus infection. Med Microbiol Immunol (2015) 204:327–34. 10.1007/s00430-015-0386-1 25648117

[121] Munks MW Gold MC Zajac AL Doom CM Morello CS Spector DH Genome-wide analysis reveals a highly diverse CD8 T cell response to murine Cytomegalovirus. J Immunol (2006) 176:3760–6. 10.4049/jimmunol.176.6.3760 16517745

[122] Nikolich-Zugich J Slifka MK Messaoudi I. The many important facets of T-cell repertoire diversity. Nat Rev Immunol (2004) 4:123–32. 10.1038/nri1292 15040585

[123] Jenkins MK Chu HH McLachlan JB Moon JJ. On the composition of the preimmune repertoire of T cells specific for peptide-major histocompatibility complex ligands. Annu Rev Immunol (2010) 28:275–94. 10.1146/annurev-immunol-030409-101253 20307209

[124] Jenkins MK Moon JJ. The role of naive T cell precursor frequency and recruitment in dictating immune response magnitude. J Immunol (2012) 188:4135–40. 10.4049/jimmunol.1102661 22517866 PMC3334329

[125] Jaeger S Fernandez B Ferrier P. Epigenetic aspects of lymphocyte antigen receptor gene rearrangement or ‘when stochasticity completes randomness’. Immunology (2013) 139:141–50. 10.1111/imm.12057 23278765 PMC3647179

[126] Holtappels R Simon CO Munks MW Thomas D Deegen P Kühnapfel B Subdominant CD8 T-cell epitopes account for protection against Cytomegalovirus independent of immunodomination. J Virol (2008) 82:5781–96. 10.1128/JVI.00155-08 18367531 PMC2395166

[127] Nauerth M Weißbrich B Knall R Franz T Dössinger G Bet J TCR-ligand koff rate correlates with the protective capacity of antigen-specific CD8+ T cells for adoptive transfer. Sci Transl Med (2013) 5:192ra87. 10.1126/scitranslmed.3005958 23825303 PMC3991308

[128] Lemmermann NA Gergely K Böhm V Deegen P Däubner T Reddehase MJ. Immune evasion proteins of murine Cytomegalovirus preferentially affect cell surface display of recently generated peptide presentation complexes. J Virol (2010) 84:1221–36. 10.1128/JVI.02087-09 19906905 PMC2812335

[129] Lemmermann NA Fink A Podlech J Ebert S Wilhelmi V Böhm V Murine Cytomegalovirus immune evasion proteins operative in the MHC class I pathway of antigen processing and presentation: state of knowledge, revisions, and questions. Med Microbiol Immunol (2012) 201:497–512. 10.1007/s00430-012-0257-y 22961127

[130] Holtappels R Thomas D Podlech J Reddehase MJ. Two antigenic peptides from genes m123 and m164 of murine Cytomegalovirus quantitatively dominate CD8 T-cell memory in the H-2d haplotype. J Virol (2002) 76:151–64. 10.1128/JVI.76.1.151-164.2002 11739681 PMC135724

[131] Däubner T Fink A Seitz A Tenzer S Müller J Strand D A novel transmembrane domain mediating retention of a highly motile herpesvirus glycoprotein in the endoplasmic reticulum. J Gen Virol (2010) 91:1524–34. 10.1099/vir.0.018580-0 20147515

[132] Fink A Büttner JK Thomas D Holtappels R Reddehase MJ Lemmermann NA. Noncanonical expression of a murine Cytomegalovirus early protein CD8 T-cell epitope as an immediate early epitope based on transcription from an upstream gene. Viruses (2014) 6:808–31. 10.3390/v6020808 24535000 PMC3939483

[133] Ebert S Lemmermann NA Thomas D Renzaho A Reddehase MJ Holtappels R. Immune control in the absence of immunodominant epitopes: implications for immunotherapy of Cytomegalovirus infection with antiviral CD8 T cells. Med Microbiol Immunol (2012) 201:541–50. 10.1007/s00430-012-0268-8 22976556

[134] Steffens HP Kurz S Holtappels R Reddehase MJ. Preemptive CD8 T-cell immunotherapy of acute Cytomegalovirus infection prevents lethal disease, limits the burden of latent viral genomes, and reduces the risk of virus recurrence. J Virol (1998) 72:1797–804. 9499030 10.1128/jvi.72.3.1797-1804.1998 PMC109469

[135] Pahl-Seibert MF Juelch M Podlech J Thomas D Deegen P Reddehase MJ Highly protective in vivo function of Cytomegalovirus IE1 epitope-specific memory CD8 T cells purified by T-cell receptor-based cell sorting. J Virol (2005) 79:5400–13. 10.1128/JVI.79.9.5400-5413.2005 15827154 PMC1082747

[136] Reddehase MJ Balthesen M Rapp M Jonjić S Pavić I Koszinowski UH. The conditions of primary infection define the load of latent viral genome in organs and the risk of recurrent Cytomegalovirus disease. J Exp Med (1994) 179:185–93. 10.1084/jem.179.1.185 8270864 PMC2191331

[137] Wills MR Mason GM Sissons JG Adaptive cellular immunity to human Cytomegalovirus. In: Reddehase MJ, editor. Cytomegaloviruses: From Molecular Pathogenesis to Intervention. (Vol. 2), Norfolk, UK: Caister Academic Press (2013). p. 141–71.

